# Digital assessment of cognitive-affective biases related to mental health

**DOI:** 10.1371/journal.pdig.0000595

**Published:** 2024-08-29

**Authors:** Sang-Eon Park, Jisu Chung, Jeonghyun Lee, Minwoo JB Kim, Jinhee Kim, Hong Jin Jeon, Hyungsook Kim, Choongwan Woo, Hackjin Kim, Sang Ah Lee

**Affiliations:** 1 Department of Brain and Cognitive Sciences, Seoul National University, Seoul, Republic of Korea; 2 Department of Biomedical Engineering, Ulsan National Institute of Science and Technology, Ulsan, Republic of Korea; 3 School of Psychology, Korea University, Seoul, Republic of Korea; 4 Department of Psychiatry, Depression Center, Samsung Medical Center, Sungkyunkwan University School of Medicine, Seoul, Republic of Korea; 5 Hanyang Digital Healthcare Center, Hanyang University, Seoul, Republic of Korea; 6 Center for Neuroscience Imaging Research, Institute for Basic Science (IBS), Suwon, Republic of Korea; 7 Department of Biomedical Engineering, Sungkyunkwan University, Suwon, Republic of Korea; 8 Department of Intelligent Precision Healthcare Convergence, Sungkyunkwan University, Suwon, Republic of Korea; The University of Hong Kong, HONG KONG

## Abstract

With an increasing societal need for digital therapy solutions for poor mental health, we face a corresponding rise in demand for scientifically validated digital contents. In this study we aimed to lay a sound scientific foundation for the development of brain-based digital therapeutics to assess and monitor cognitive effects of social and emotional bias across diverse populations and age-ranges. First, we developed three computerized cognitive tasks using animated graphics: 1) an emotional flanker task designed to test attentional bias, 2) an emotional go-no-go task to measure bias in memory and executive function, and 3) an emotional social evaluation task to measure sensitivity to social judgments. Then, we confirmed the generalizability of our results in a wide range of samples (children (N = 50), young adults (N = 172), older adults (N = 39), online young adults (N=93), and depression patients (N = 41)) using touchscreen and online computer-based tasks, and devised a spontaneous thought generation task that was strongly associated with, and therefore could potentially serve as an alternative to, self-report scales. Using PCA, we extracted five components that represented different aspects of cognitive-affective function (emotional bias, emotional sensitivity, general accuracy, and general/social attention). Next, a gamified version of the above tasks was developed to test the feasibility of digital cognitive training over a 2-week period. A pilot training study utilizing this application showed decreases in emotional bias in the training group (that were not observed in the control group), which was correlated with a reduction in anxiety symptoms. Using a 2-channel wearable EEG system, we found that frontal alpha and gamma power were associated with both emotional bias and its reduction across the 2-week training period.

## Author summary

This study aimed to provide a scientific basis for digital therapeutic tools assessing and monitoring affective and cognitive health using user-friendly tasks. We developed three emotional cognitive tasks (eFlanker, eGoNoGo, and eSocial) on a wide range of participants (children, young adults, older adults, and depression patients). Abstract scores were extracted from a large set of behavioral features such as reaction time and accuracy to positive, negative, and neutral animated face stimuli; a subset of these scores were found to be strongly associated with emotional bias and mental health. Two weeks of training with gamified versions of the tasks decreased the emotional bias, which was also related to the reduction of anxiety symptoms. Using a wearable EEG system, we found that changes in emotional bias after training were related to changes in frontal alpha and gamma activity. These results demonstrated the potential effectiveness of digital gamified applications for the monitoring and reduction of emotional bias in cognition, which was related to mental health measures such as anxiety and depression.

## Introduction

Despite the rise in public awareness of the importance of mental health, rates of depression and anxiety remain extremely high [1–3]. One of the underappreciated consequences of poor affective health is its detrimental effects on cognition [4], which perpetuate the vicious cycle of underperforming in one’s education or career and a low sense of well-being [5]. Cognitive psychologists and neuroscientists have shown time and time again that cognitive functions such as emotional processing, attention, executive function, and memory are impaired in individuals with anxiety or depression [3,6], and that these impairments are associated with functional abnormalities in the brain’s fronto-limbic network [7–10]. Unfortunately, tests to assess such impairments have mostly remained limited to self-report survey items (e.g., on difficulty concentrating and mental fogginess) or to the confines of the laboratory, making them neither easily accessible nor sustainable for use by the general public. In the emerging field of digital therapeutics, therefore, one of the remaining challenges for both researchers and clinicians is to develop a tool to objectively monitor and potentially improve the interaction between cognition and affect that can be used by individuals across a wide age range.

Most digital therapeutics tools currently being developed to tackle affective health involve explicit simulation games for cognitive behavioral therapy, meditation, or self-assessment-based record-keeping of one’s own state [11,12]. On the other hand, “serious games” have been successfully applied to evaluate and improve impairments in “cold” cognition [13,14] such as attention and memory [15–17]. Unfortunately, despite the overwhelming evidence that mental health is influenced by biases in emotional, social, and cognitive processing [18–21], there is still a lack of digital tools that capture such interactions within the scope of a game-like application that can be widely used by the public. This is especially challenging due to the fact that emotional digital contents for fast-paced cognitive tasks are difficult to create and validate. In laboratory experiments, emotional responses are most often elicited using photographs of emotional expressions (e.g., angry, happy, fearful faces) [22] or scenes containing violent or graphic images [23,24]. However, such contents are inappropriate for usage in gamified tools and potentially harmful to vulnerable populations at high risk for affective disorders.

Another challenge to creating digital therapeutics for cognitive-affective health is that purely behavioral measures are met with uncertainty about their underlying neural mechanisms. While scientifically it would be more precise to measure neural activity while people are performing the cognitive tasks, that option has its own set of impracticalities from the perspective of developing at-home self-administered digital therapeutics applications. Wearable EEG headsets that measure frontal lobe activity have been commercially developed for various purposes, ranging from meditation, sleep-aids, to cognitive training. Given the existing evidence that relates prefrontal resting-state EEG activity to mental health [25–28] and cognition [29–32], a frontal EEG biomarker of cognitive-affective health that can be measured with a simple wearable system may be instrumental for the advancement of brain-related digital technology.

### The present study

**Fig 1:**
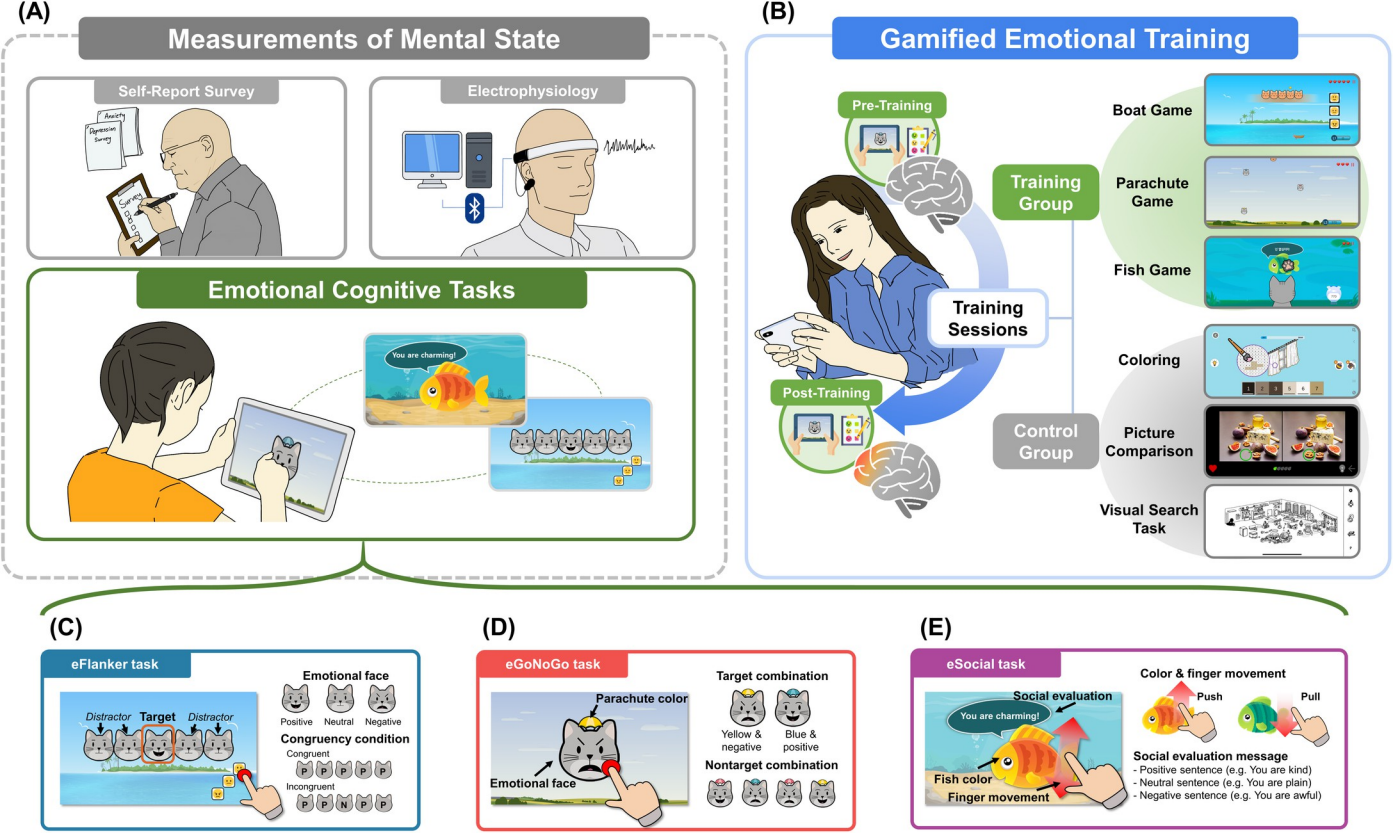
The overall paradigm of this study. (A) There were three assessments of neurocognitive state: self-report survey, electrophysiology, and emotional cognitive tasks. (B) Emotional training consisted of three digital games similar to the emotional cognitive tasks (Boat, Parachute, and Fishing games). The control task consisted of 3 non-emotional games (coloring, picture comparison, and visual search tasks. (C-E) A diagram with an example screenshot of the emotional cognitive tasks: (C) eFlanker, (D) eGoNoGo, and (E) eSocial tasks.

In order to fulfill the various unmet needs in the development of cognitive digital therapeutics, we created a series of computerized, touch-screen tasks (**Fig 1**) inspired by past studies on cognitive impairments in anxiety and depression, particularly those involving emotional stimuli [33–36], with the intention of developing a digital assessment appropriate for users of all ages (**Fig 1C–1E**). The tasks consisted of an emotional flanker task (eFlanker), an emotional go-no-go task (eGoNoGo), and an emotional social evaluation task (eSocial) [37], designed to measure participants’ cognitive and emotional processing, using simple emoticon-like graphics. Additionally, we explored the validity of the spontaneous thought generation task [38,39] as a potential alternative to self-report scales of mental health. We hypothesized that individuals with higher anxiety and depression would show more difficulty and bias in processing emotional information, especially in tasks that are cognitively demanding, and aimed to identify multiple measures that correlated with individuals’ mental health (e.g., anxiety, depression, and self-esteem).

After confirming the validity of our tasks in 172 young, healthy Korean adults (age 19–38), we performed follow-up experiments with a subset of the tasks in depression patients (N = 41, age 20–67), young children (N = 50, age 5–9), older adults (N = 39, age 50–75), and online participants in the U.S. and U.K. (N = 93, age 19–37) to further test the generalizability of the results across a wide population. Given the inter-correlation among many of the behavioral reaction times and accuracy scores obtained from the cognitive tasks, we converted them into abstract features (G-scores) which captured the cognitive-affective characteristics of reduced attention and emotional bias in individuals with higher anxiety and depression [18,34,40] as well as atypical emotional-social processing associated with lower self-esteem [41,42].

We further tested the hypothesis that a gamified training tool consisting of the above cognitive tasks can improve one’s ability to regulate the biased processing of emotional stimuli. Using the above contents, we created a phone-based game inspired by the above cognitive tasks and conducted a 2-week training study on 71 of the original younger adult sample (**Fig 1B**). We found training effects on attention indicated by shortened RT and, more importantly, on emotional bias which mediated the alleviation of anxiety symptoms.

To support our behavioral findings with neural measures that can be utilized for brain-based digital therapy, we collected resting-state neural activity from a subset of participants using a wearable 2-channel frontal lobe EEG system. To directly associate individual cognitive-affective bias with brain activity, we identified neural markers associated with emotional bias and mental health, as well as their change across training.

## Methods

### Ethics

This study was conducted according to the Declaration of Helsinki, and all experimental procedures were approved by the institutional review boards for human research (IRB) of Seoul National University (SNU), Korea University (KU), Sungkyunkwan University (SKKU), Hanyang University (HYU), Hanyang University Hospital (HYUH), and Samsung Medical Center (SMC) (Approval No. SNU: 2202/003-017 (young and older adults), 2205/001-012 (training), 2201/003-006 (children), KU: 2022-0049-01, SKKU: 2022-01-025, HYU: 202303-026-3, HYUH: 2022-07-017, and SMC: 2022-05-117). Written informed consent was obtained from all participants or from the parent/guardian of participants for children under 18 years of age.

### Participants

We collected data from five different samples across various settings: offline young adults, patients, children, older adults, and online young adults (Table 1, no overlap across sample groups). The sample for our main analysis was the offline young adult group, as they performed every emotional cognitive task, and the other samples were used to test the generalizability of our findings and sometimes performed only a subset of the behavioral, affective and EEG measures that were collected in the offline young adults (see detailed description below).

#### Offline young adults

Participants between 20 and 40 years of age (mean = 24.2 and Std = 4) were recruited from the local community (N = 172, 98 females) and were compensated with approximately 15 USD for 45 minutes of participation. The compensation was slightly higher per hour for offline patients, children, and older adults compared to the young adult groups based on their additional transportation expenses. 71 of them also participated in the training study (Training group: N = 43, 21 females, Age: mean = 25 and Std = 4.1; Control group: N = 28, 12 females, Age: mean = 23.2 and Std = 4.3). All participants had corrected-to-normal or normal vision, were currently attending or had received college level education, and had no history of mental illness. Depression, anxiety, and self-esteem were measured using the following scales: Patient Health Questionnaire-9 Item (PHQ-9 [43]), Center for Epidemiological Studies-Depression Scale (CES-D [44]), Beck’s depression inventory (BDI-II [45,46]), Spielberger’s State-Trait Anxiety Inventory Form Y (STAI-YS and STAI-YT [47]), and Rosenberg Self-Esteem Scale (RSES [48,49]). 101 of the participants also completed the spontaneous thought generation task [38,39].

#### Patients

The major depressive disorder (MDD) patients diagnosed based on DSM-5 [50] (mean age = 45 and Std = 12.2) were recruited from Hanyang University Hospital and Samsung Medical Center (N = 41, 36 females) and compensated with approximately 15 USD for 30 minutes of participation. The patients performed a shorter version of the three tasks (eFlanker: reduced from 270 to 144 trials; eGoNoGo: reduced from 240 to 140 trials; eSocial: reduced from 204 to 114 trials; see S1 Table for the trial number for each condition and see *Measurement of Cognition and Affect* section for a detailed description of the cognitive tasks) to reduce the overall length of the session. The number of practice trials was not changed. For the eFlanker and eGoNoGo tasks, the trial number was shortened while maintaining the ratio between the conditions of the task (e.g. eFlanker ‐ 30 trials to 16 trials per each condition). For the eSocial task, the emotional target condition was reduced by 40% and the non-target condition was 56%. Depression was measured using the PHQ-9 scale. 34 of the participants also completed the spontaneous thought generation task.

#### Children

Children between 5 to 9 of age without any history of mental illness were recruited from a local community center (N = 50, 26 females, Age: mean = 6.7 and Std = 1.2) and were compensated with approximately 15 USD for 30 minutes of participation (based on their additional transportation expenses). The number of practice trials were halved for both eFlanker and eGoNoGo tasks. For the eFlanker and eGoNoGo tasks, the trial number was shortened while maintaining the ratio between conditions (e.g. eFlanker ‐ 30 trials to 20 trials per condition, see S1 Table for the trial number). Anxiety and depression were measured using the STAI and CES-D scales for children (STAI-CH [51] and CES-DC [52]).

#### Older adults

Older adults between 50 and 74 of age (mean = 62.8 and Std = 6.0) without any history of mental illness were recruited from a local community center and Hanyang Digital Healthcare Center (N = 39, 26 females) and were compensated with approximately 15 USD for 30 minutes of participation (based on their additional transportation expenses). The older adults only performed the eFlanker task. Anxiety, depression, and self-esteem were measured using the STAI-YT, PHQ-9, CES-D, and RSES scales. 16 of the participants also completed the spontaneous thought generation task. Only participants who reported PHQ-9 scale under 10 were recruited.

#### Online young adults

A total of 93 U.K. and U.S. participants (46 females) between 20 and 40 of age (mean = 28.6 and Std = 6.4) were recruited online through Prolific (Prolific, U.K.) and were compensated with 10 USD for 45 minutes of participation. All participants had corrected-to-normal or normal vision, were either in college or received college-level education, and had no history of mental illness. Participants performed the task using a desktop computer and keyboard. Anxiety and depression were measured using the STAI-YS, STAI-YT, PHQ-9, and CES-D scales.

**Table 1:** Participant information and the cognitive tasks that they performed.

	N	Age (mean±std)	Sex	PHQ-9^a^	CES-D^a^	BDI-II ^a^	STAI-YT^a^	STAI-YS^a^	RSES^a^	Tasks performed
Offline young adults	172	24.2±4	M:74 F:98	5.6 (0, 21)	15.1 (0, 40)	12.5 (0, 40) (N = 131)	43.8 (20, 70)	43.4 (20, 73)	29.1 (15, 40) (N = 131)	eFlanker eGoNoGo eSocial STG^c^
Patients	41	45±12.2	M:5 F:36	15.1 (10,19)	Not collected	Not collected	Not collected	Not collected	Not collected	eFlanker^d^ eGoNoGo^e^ eSocial^f^
Children	50	6.7±1.3	M:24 F:26	Not collected	14.4^b^ (0,44)	Not collected	31.1^b^ (20,46)	Not collected	Not collected	eFlanker^g^ eGoNoGo^h^
Older adults	39	62.8±6	M:13 F:26	2.8 (0, 9)	11.9 (0, 25) (N = 23)	11.3 (0, 38) (N = 21)	35.9 (20, 47) (N = 18)	Not collected	26.3 (0, 40) (N = 23)	eFlanker STG^c^
Online young adults	93	27.8±5.4	M:47 F:46	18.0 (9, 31)	21.8 (2, 47)	Not collected	47.7 (21, 75)	43 (20, 76)	Not collected	eFlanker eGoNoGo

^a^: mean (min, max)

^b^: children’s version (CES-DC and STAI-CH)

^c^: Spontaneous thought generation task

^d, e, f^: a shorter version for patients (see Methods and S1 Table for details)

^g, h^: a shorter version for children (see Methods and S1 Table for details)

### Measurement of Cognition and Affect

#### Emotional cognitive tasks

We designed three cognitive tasks (eFlanker, eGoNoGo, and eSocial) to measure individual inhibition, emotional processing, and selective attention (**Fig 1A and 1C–1E**).

1. *Emotional flanker task (eFlanker)*: The eFlanker was designed to test for biases in emotional attention [53,54]. Participants were instructed to choose the face expressing the same emotion as the target which was presented in the middle, with two distractors on each side. The stimulus was presented after a brief fixation cross (1000ms) and, once the participant responded, feedback was provided on the screen. The target and distractor consisted of positive (smiling), neutral, and negative (frowning) animated cat faces, and their presentation as the target and distractor comprised a total of 9 possible combinations. After a practice block of 36 trials (for familiarization with the task), participants performed a total of 270 test trials (30 trials for each condition), presented in random order.
2. *Emotional Go/No-Go task (eGoNoGo)*: The eGoNoGo task was designed to measure emotional bias in executive function and memory, in order to avoid choosing the non-target objects [55,56]. Out of 6 possible combinations from 3 parachute colors (yellow, red, and blue) and 2 emotions (positive and negative), participants were asked to respond to two target combinations of a specific parachute color and a specific emotional cat face (e.g. positive cat face with the blue parachute and the negative cat face with the yellow parachute). The color-emotion combination was counterbalanced across participants. After the fixation cross was presented for 1000ms, the non-target trial was presented for 1500ms and the target trial was presented until the participants responded. If participants responded to the non-target (incorrect response) or responded to the target later than 1500ms (for offline young adults, Patients, and Online young adults) or 2000ms (for children), visual feedback (“Incorrect” or “Respond quickly”) was displayed for 1000ms. After a practice block of 40 trials, participants performed a total of 270 test trials. We adjusted the frequency of the target trials to (40% of the total trials to offset the imbalance between the target (2 out of 6 possible combinations) and non-target trials (4 out of 6) to keep the participants’ inhibitory attention engaged [57,58].
3. *Social approach-avoidance task (eSocial task)* [37]: We designed the eSocial task to measure sensitivity in interpersonal behavior by observing the approach or avoidance behavior in response to an evaluative message [59,60]. Critically, the target was concurrently presented with a message conveying negative or positive social evaluations about the participant. After the brief fixation period (1000ms), the participant was asked to respond with pulling (approach) or pushing (avoidance) finger movements based on the color of a centrally presented target (e.g., yellow = push or green = pull), in the form of an animated fish character with the evaluative comment (e.g., “You are selfish.”) in a speech bubble next to it. In the case of the non-target condition, the social evaluation message was replaced by an instruction message (“Take me with you” or “Send me away”) for the participant to push or pull the fish regardless of its color. Participants were given an unlimited time to respond. After a practice block of 4 trials, participants performed a total of 204 test trials (150 trials with 60 positive, 60 negative, and 30 neutral; 54 non-target trials), with a balanced number of approach (pull) or avoidance (push) trials.

#### Spontaneous thought generation task

In the spontaneous thought generation task [38,39] (S1 Fig), participants were first instructed to freely think during the 30 seconds of the fixation period. Then, they were given an unlimited time to type one word that came to mind. Finally, participants were asked to rate the self-relevance (from irrelevant to relevant) and valence (from negative to neutral and positive) on a continuous scale using a moving bar. Total 15 trials were conducted (i.e., 15 words).

#### EEG measurement

To investigate the electrophysiological markers and neurobiological mechanisms underlying individual differences in behavior related to affective and cognition, we recorded EEG signals from a total of 71 young adults over a three-minute resting-state period with their eyes closed. A wireless 2-channel EEG headband (LAXTHA Inc., nnFX2) was used by consideration of its potential applicability in digital therapeutic tools. The headband was placed across the forehead, with electrodes positioned approximately at AF7 and AF8. A clip-type earlobe electrode served as the reference electrode.

### Procedures

#### General procedures

At the start of the session, participants were provided with informed consent and completed the self-report surveys. EEG data collection took place next, while participants were in a seated position with their eyes closed, followed by the spontaneous thought generation task. Cognitive tasks were administered last, in a mixed order. Note that different samples of participants performed a subset of the above procedures (see Section *Participants* for details).

#### Emotional digital game-based training

1. **Procedures of the training study:** The emotion training or the control task was performed 4 (training group N = 22, control N = 19) or 6 (training N = 21, control N = 9, please see details of the training and control tasks below) times within two weeks by using a cell phone under surveillance (**Fig 1B**). Total 71 (training group N = 43 and control group N = 28) participants conducted the emotional cognitive tasks before and after the training (or the control task), and we characterized training effects by comparing behavioral indices as well as their association with affective scale and neural changes. Affective scales by self-reported surveys and resting-state EEG data (3 min) were also collected before conducting the emotional cognitive tasks
2. **Gamified digital game-based training:** The emotional training consisted of three games similar to the emotional cognitive tasks: The Boat game involved choosing the facial expression that matched the target cat’s face (**Fig 1B** and S1 Video). The target and four additional distractors of the cat face with different emotions gradually fell from the top of the screen together, and the target was indicated by a star. If a response was made before the cats hit the water, the distractor cats disappeared and the emotional face of the target cat changed. If the cat was a smiling cat, participants had to rescue it by dragging and placing the boat under it. The Parachute game asked participants to touch (and eliminate) the cat face that did not match with the target combinations of facial expression and parachute color (**Fig 1B** and S1 Video). Among six combinations of parachute colors (red, blue, and yellow) and cat facial expressions (angry or happy), the targets were an angry facial expression with one color parachute (e.g. red) and a happy face with a different color of the parachute (e.g. yellow). The target colors were randomly selected at the beginning of the game. Third, in the Fish game, the participants were asked to push or pull the fish with their index finger based on its color (**Fig 1B** and S1 Video). Occasionally, the speech bubbles made by the fish did not include social evaluation contents but instructed participants to push or pull the fish regardless of its color, which made it necessary for participants to read the speech bubbles. Every training game consisted of 5 difficulty levels by adjusting the time limit to respond (Boat, Parachute, and Fish), the number of faces falling (Parachute), and the number of trials (Fish). The control task consisted of 3 non-emotional games (coloring, picture comparison, and visual search task, **Fig 1B**). Each training or control session consisted of all three games, and the duration of one game was about 10 minutes (a total of 30 minutes for one session).

### Calculation of Behavioral and Neural Markers

#### Behavioral indices of the emotional cognitive tasks

Reaction time (RT) and accuracy were calculated from the emotional cognitive tasks. We averaged RTs from the correct trials, removing those shorter than 200ms to eliminate accidentally pressed responses. Outliers for RT or accuracy in each emotional condition were excluded at the subject level.

#### Behavioral indices of the spontaneous thought generation task

We analyzed two behavioral indices, self-relevance and valence ratings, from the spontaneous thought generation task [38,39]. Self-reported values were normalized to be ranged from -1 (irrelevant or negative) to +1 (relevant or positive), and the two indices were averaged across the 15 trials.

#### Calculating G-scores as latent factors of behavioral indices

Low-dimensional features were needed to describe a range of features in the participants’ performance on the emotional cognitive tasks. First, we extracted 129 performance features consisting of RT and accuracy from various conditions of the three tasks (eFlanker, eGoNoGo, and eSocial) ‐ for example, averaged RT/accuracy of whole trials or RT/accuracy difference between emotional conditions (e.g. RT difference between negative and positive conditions). Task performance of healthy young adults (N = 172, identical to the previous section) and depression patients were used for the analysis to include a wide range of behavioral variation. Second, low-dimensional latent variables of task performance were generated by PCA after z-scoring every feature [61,62]. To minimize the over-representation of outliers during PCA, features with an absolute value over 2.5 were downregulated to 2.5. The K-means clustering method was applied to the two principal components for dividing the latent variables into several groups based on the squared Euclidean distance [63,64]. Performance features within the same group represented a similar pattern across the participants (e.g. RT in the eFlanker negative condition was highly correlated with RT in the eFlanker neutral condition). The post hoc silhouette analysis verified that 5 was the optimal number to maximally separate the latent variables. Lastly, we calculated G-scores 1 to 5 by calculating the major PC from each of the 5 feature groups after z-scoring every feature.

#### EEG preprocessing and flattened power spectral density calculation

EEG signals were collected at a 250 Hz sampling rate and bandpass filtered between 0.1 and 50Hz. We rejected 6 participants based on visual inspection of noisy signals. To remove artifacts in the EEG signal, we employed artifact subspace reconstruction from the EEGLAB toolbox [65], an automated technique for detecting and removing transient high-amplitude artifacts. We then computed the power spectral density (PSD) in a sliding 10-second moving window with a step size of 2.5 seconds. We then applied the FOOOF algorithm (fitting oscillations & one-over-f, see [66] for methodological details) to decompose the aperiodic exponent (i.e., spectral slope between log-frequency and log-PSD) and the periodic narrowband power (i.e., residuals by subtracting the spectral slope fitting from the original log-PSD) [67–69]. We then averaged the aperiodic exponent or band power (theta: 4-8Hz, alpha: 8-12Hz, and gamma: 25-40Hz) from all windows.

#### Effects of the emotional digital training

G-scores between pre- and post-training sessions were compared. In addition, a correlation between changes in G-scores and in the flattened band power (theta, alpha, and gamma) was measured to connect the changes in affective scales, G-scores, and neural activity. We compared the control and experimental groups to look for specific effects of the digital emotional training.

## Results

### The battery of emotional cognitive tasks

The emotional cognitive tasks (eFlanker, eGoNoGo, and eSocial) were performed by young adults (N = 172, age 19–38) in the laboratory environment. We analyzed RT and accuracy differences across various emotional conditions and their relation to mental health measures.

#### eFlanker task

**Fig 2:**
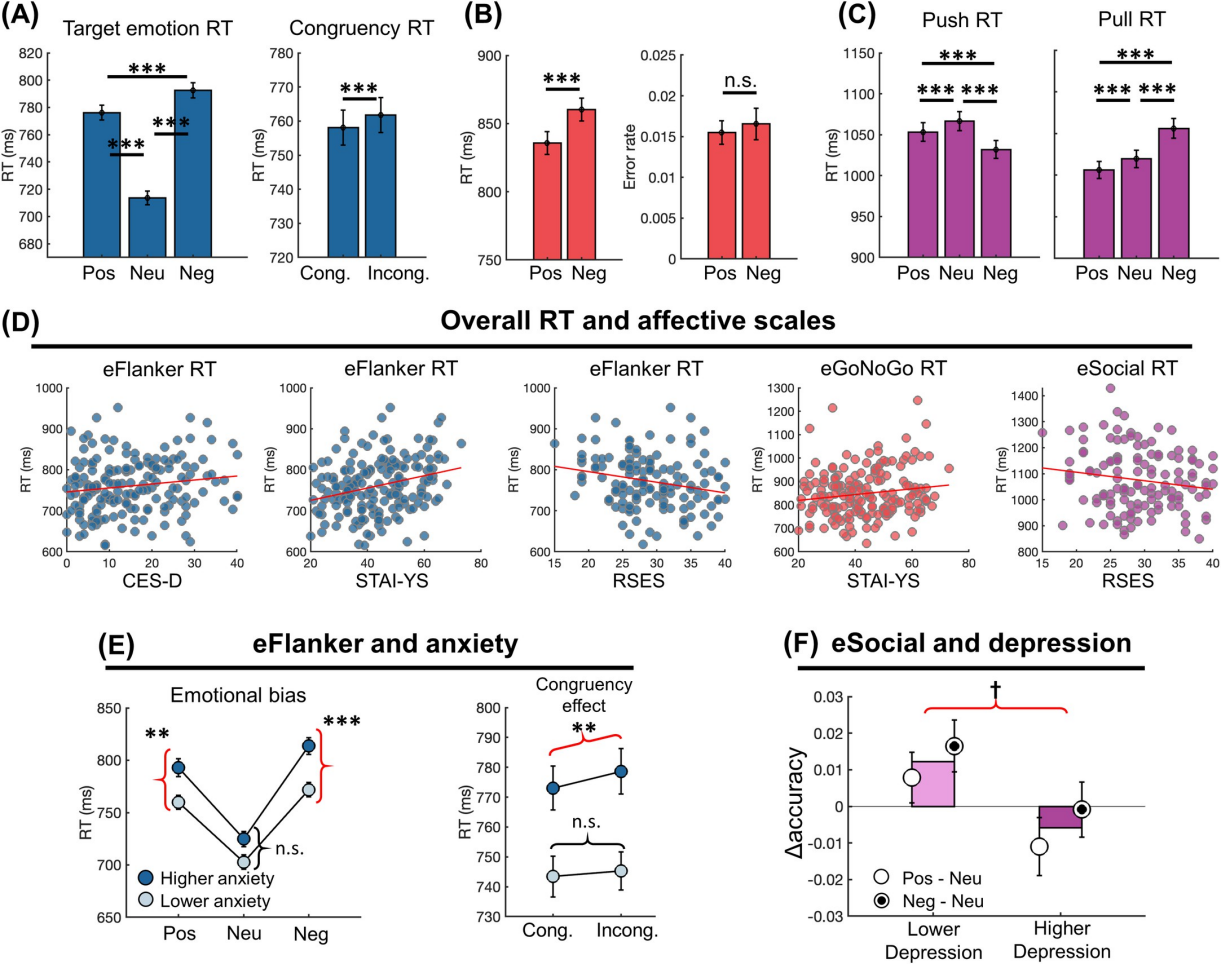
Behavioral results from young adults in the three emotional cognitive tasks. **(A)** A comparison of RT from the eFlanker task across three emotional conditions or congruent/incongruent conditions. **(B)** RT and error from the eGoNoGo task in the positive and negative conditions. **(C)** A comparison of RT from the eSocial task in the push and pull conditions across three emotions. **(D)** A significant correlation between RT from the three tasks and depression (CES-D), state anxiety (STAI-YS), self-esteem scale (RSES). **(E)** The higher anxiety group (median split) showed higher RT in the emotion conditions (left) and a larger congruency effect (right). **(F)** The lower depression group (median split) showed a larger accuracy difference between emotional (positive and negative) and neutral conditions in the eSocial task. Error bars indicate standard error. ^†^: p<0.1, *: p<0.05, **: p<0.01, ***: p<0.001.

A repeated measures ANOVA (RMANOVA) was performed on the RT of the eFlanker task with sex as the between-subjects variable and target emotion and congruency as within-subjects variables. We found main effects of the target emotion (F(2,340) = 367.062, p<0.001) and congruency (F(1,170) = 7.242, p = 0.008). RT was slowest in the negative condition followed by the positive and neutral conditions (**Fig 2A**, positive vs. neutral: t(171) = 19.401, p<0.001; positive vs. negative: t(171) = -6.335, p = 0.001; negative vs. neutral: t(171) = 23.479, p<0.001, *uncorrected*) and slower in the incongruent condition than the congruent condition (**Fig 2A**, congruent vs. incongruent: t(171) = -2.893, p = 0.004).

Next, we added each affective scale (depression: CES-D; anxiety: STAI-YS; self-esteem: RSES) into the ANOVA to assess its interactions with the behavioral indices. First, all affective scales showed a main effect (CES-D: F(1,169) = 3.837, p = 0.052; STAI-YS: F(1,169) = 14.338, p<0.001; RSES: F(1,169) = 5.878, p = 0.017), with slow RT associated with lower self-esteem (**Fig 2D**, Pearson r = -0.225 and p = 0.010), higher anxiety (**Fig 2D**, r = 0.281, p<0.001) and depression (**Fig 2D**, r = 0.151, p = 0.048). We divided subjects into low and high groups based on a median split on the respective mental health scales and found an interaction between anxiety level and target emotion (F(2,336) = 6.151, p = 0.002). Participants with higher anxiety were slower, particularly in response to emotional targets (**Fig 2E**, low vs. high anxiety in *positive*: t(170) = -3.062, p = 0.009; *neutral*: t(170) = -2.206, p = 0.087; *negative*: t(170) = -3.946, p<0.001, *Bonferroni corrected*), revealing their difficulties in processing emotional information in general. This pattern was not observed in participants with higher depression (S2 Fig)

Furthermore, depression and anxiety scales also showed an interaction with *congruency* (CES-D: F(1,169) = 5.847, p = 0.017; STAI-Ys: F(1,169) = 4.571, p = 0.034). The higher depression and anxiety groups showed significantly higher RT in incongruent than congruent conditions (**Fig 2E**, congruent vs. incongruent in the higher anxiety group: t(84) = -2.872, p = 0.005; S2 Fig, in the higher depression group: t(78) = -2.529, p = 0.013, *uncorrected*), while groups with lower anxiety or depression did not show any pronounced congruency effect (**Fig 2E**, congruent vs. incongruent in the lower anxiety group: t(86) = -1.114, p = 0.268; S2 Fig, in the lower depression group: t(92) = -1.539, p = 0.127, *uncorrected*).

Lastly, a three-way interaction effect on RT (interaction across congruency, emotion, and depression: F(2,338) = 3.921, p = 0.021; anxiety: F(2,338) = 4.136, p = 0.017) indicated that a congruency effect varied across the target emotion types in the higher depression/anxiety group, while the lower depression/anxiety group showed similar congruency effects across all emotional conditions (S2 Fig). In particular, the higher depression/anxiety group showed a higher congruency effect in the positive condition but a lower congruency effect in the negative condition compared to the lower depression/anxiety group.

Overall, these results indicated that individuals with higher depression or anxiety suffered from impaired attention while emotional attention bias was more pronounced in the higher anxiety group. The accuracy was not significantly different across conditions, affective scales, and their interactions.

#### eGoNoGo task

An RMANOVA was performed on both RT and accuracy of the eGoNoGo task with sex as the between-subjects variable and target emotion as the within-subjects variable. The target emotion had a significant main effect on RT (**Fig 2B**, F(1,170) = 26.309, p<0.001), with RT in the negative condition slower than the positive condition. We also found a main effect of anxiety scales on RT (F(1,169) = 3.964, p = 0.048), with higher anxiety participants showing a higher RT (**Fig 2D**, r = 0.146, p = 0.055). The other affective scales such as depression and self-esteem did not show significant effects on RT. The accuracy was not significantly different across conditions, affective scales, and their interactions.

#### eSocial task

The push and pull conditions of the eSocial task were designed to assess the control ability of social avoidance and approach, respectively. RTs and accuracies in the push and pull conditions were separately tested by an RMANOVA with sex as the between-subjects variable and emotion (positive, neutral, and negative) as the within-subject variable. In both conditions, emotion showed a significant main effect on RT (**Fig 2C**, push: F(2,340) = 33.978, p<0.001; pull: F(2,340) = 69.674, p<0.001). Post hoc tests in the push condition (i.e. avoidance) showed that RTs were fastest for negative social evaluations followed by the positive and then neutral evaluations (positive vs. neutral: t(171) = -3.230, p = 0.004; positive vs. negative: t(171) = 6.325, p<0.001; negative vs. neutral: t(171) = -7.365, p<0.001, *Bonferroni corrected*). On the other hand, in the pull condition (i.e. approach), RTs were slowest for the negative evaluations followed by neutral and then positive evaluations (positive vs. neutral: t(171) = -4.043, p<0.001; positive vs. negative: t(171) = -10.557, p<0.001; negative vs. neutral: t(171) = 7.937, p<0.001, *Bonferroni corrected*). These results indicated that the executive function is affected by the socially evaluative messages.

Next, we again systematically added each affective scale into the RMANOVAs. First, a main effect of RSES on RTs found in the push and the pull conditions (push: F(1,169) = 4.311, p = 0.04; pull: F(1,169) = 2.987, p = 0.086) showed that participants with higher self-esteem were generally faster in their response (**Fig 2D,** push: r = -0.188, p = 0.032; pull: r = -0.167, p = 0.056). Second, the higher depression group showed lower accuracy in response to emotional evaluations than neutral ones, compared to the lower depression group (**Fig 2F,** an interaction between emotion and CES-D: F(2,338) = 3.346, p = 0.036). Lower performance in the emotional conditions in depressive individuals implicated their biased emotional processing toward social evaluation.

### Spontaneous thought generation task as a potential alternative to affective scales

During the spontaneous thought generation task [38,39], participants were asked to think freely, report what was on their mind, and rate the self-relevance and valence of their generated thought (see Methods for details). We assessed the correlation between the two behavioral indices (i.e., self-relevance and valence) and mental health measures. First, individuals who generated more self-relevant spontaneous thoughts were more depressed (S3 Fig, PHQ-9: Pearson r = 0.218, p = 0.007) but not anxious (STAI-YT: r = -0.051, p = 0.611). Additionally, individuals with lower valence ratings of their spontaneous thoughts showed higher depression (S3 Fig, CES-D: r = -0.415, p<0.001), higher anxiety (STAI-YT: r = -0.440, p<0.001), and lower self-esteem (RSES: r = 0.341, p<0.001). Based on these strong correlations and their repeatability (compared to self-report surveys), the spontaneous thought generation task has substantial advantages as an alternative or secondary measure in predicting and monitoring individual affective states in the context of a digital application.

### Generalization to a wider population and environment

We tested the generalizability of the emotional cognitive tasks by applying them to a wider range of participants including children (N = 50, age 5–9, mean 6.7), older adults (N = 39, age 50–75, mean 62.8), and depression patients (N = 41, age 19–64, mean 45). Additionally, an online experiment was conducted with young adults (Prolific, U.K.) (N = 93, age 19–37, mean 27.8) for the purposes of both international generalizability and validation using a computer keyboard-based response modality.

#### Depression patients

**Fig 3:**
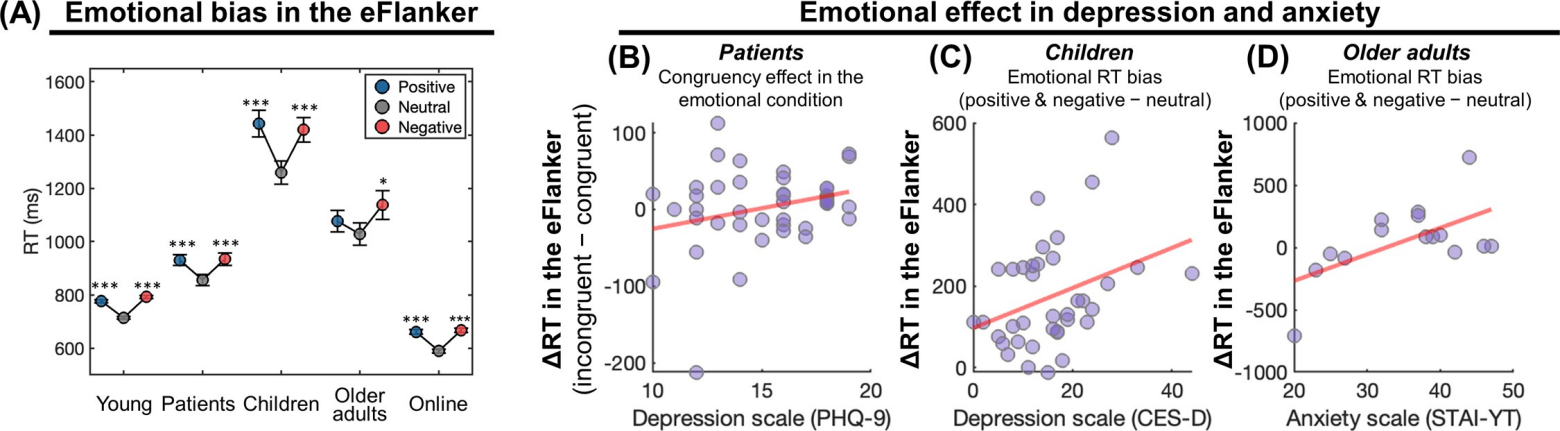
Generalized results from the eFlanker task across young adults, depression patients, younger children, older adults, and online participants. **(A)** RT was slower in the emotional (positive and negative) condition compared to the neutral condition. **(B-D)** An interaction between emotional bias and mental health. **(B)** In the patient group, a positive correlation was observed between depression (PHQ-9) and the congruency effect in the emotional condition. **(C)** In children, an emotional bias in RT was positively correlated with depression (CES-D). **(D)** In the older adult group, emotional bias in RT was positively correlated with anxiety (STAI-YT).

An RMANOVA on the eFlanker task showed a main effect of target emotion (**Fig 3A,** F(2,80) = 27.420, p<0.001), which remained significant after accounting for age as a covariate. No main effect or interaction was found when we added the sex factor in the ANOVA. Post hoc tests showed that RT was longer in the negative and positive conditions than the neutral condition (positive vs. neutral: t(40) = 6.215, p<0.001; positive vs. negative: t(40) = -0.277, p = 0.783; negative vs. neutral: t(40) = 7.402, p<0.001), reflecting the emotional processing difficulty in depression patients. Consistent with the findings from the young subclinical sample, depression severity (PHQ-9 scale) showed a significant interaction with target emotion in the congruency effect (i.e. incongruent ‐ congruent RT, F(2,78) = 3.598, p = 0.032) in that a larger congruency effect in the emotional conditions (positive and negative) was associated with depression severity (Pearson’s r = 0.308, p = 0.026, controlling for age; **Fig 3B**).

In addition, we observed a main effect of emotion on RT and accuracy in the eSocial task (push RT: F(2,74) = 7.988, p<0.001; pull RT: F(2,76) = 2.912, p = 0.06; push accuracy: F(2,74) = 5.171, p = 0.008; pull accuracy: F(2,76) = 2.546, p = 0.085). As expected the negative condition was associated with a greater tendency for avoidance (i.e. faster RT and higher accuracy in the push condition) (negative vs. neutral: t(38) = 3.860, p = <0.001) and the opposite trend for approach behavior (pull condition) (negative vs. neutral: t(38) = -1.929, p = 0.061). Consistent results from the young adults and patients showed that these tasks are sensitive to the emotional bias associated with mental health measures. No main effects or interactions were found in the eSocial task when we added sex as a factor in the ANOVA.

#### Children

Out of the 50 children who performed the eFlanker and eGoNoGo tasks, 13 and 8 children, respectively, did not finish the task and were not considered for further analyses. In general, older children responded more quickly in the eFlanker task (RT: F(1,35) = 32.863, p<0.001) and more quickly and accurately on the eGoNoGo task (RT: F(1,40) = 6.159, p = 0.017; error: F(1,40) = 5.226, p = 0.028).

Similar to the young adults, we found a main effect of target emotion (**Fig 3A,** F(2,72) = 38.095, p<0.001), with longer RTs in the positive/negative conditions than the neutral condition (positive vs. neutral: t = 8.068, p<0.001; negative vs. neutral: t = 6.92 p<0.001; positive vs. negative: t = 1.148, p = 0.764, *Bonferroni corrected*). There was no effect of sex. In addition, an interaction between target emotion and depression (CES-DC) (F(2,70) = 3.593, p = 0.033) showed that depression symptoms were significantly associated with the emotion-related slowing of RT (compared to the neutral condition) (**Fig 3C**, Pearson r = 0.342, p = 0.038).

In the eGoNoGo task, older children showed faster RT and a lower error rate (Pearson correlation between age and RT: r = -0.363, p = 0.018, error rate: r = -0.340, p = 0.028). There was no effect of sex. Similar to the young adults, anxiety (STAI-CH) was associated with slower RT (r = 0.359, p = 0.021). All effects were still significant after controlling for age.

#### Older participants

From the 39 older participants who performed the eFlanker task, 7 participants whose overall RT was longer than 2000 milliseconds or whose overall accuracy was less than 0.8 were excluded from further analysis. Consistent with the findings from the young adults, a main effect of target emotion was found in the RMANOVA test (**Fig 3A**, F(2, 62) = 3.768, p = 0.029). There was no effect of sex when it was included as a factor. Post hoc tests indicated that RT in the negative/positive conditions was slower than the neutral conditions (positive vs. neutral: t(31) = 1.143, p = 0.262; negative vs. neutral: t(31) = 2.637, p = 0.013, *not corrected*). The slowing of RT in the emotional conditions was associated with higher anxiety (**Fig 3D,** Pearson r = 0.599, p = 0.018 from the 15 participants who completed the STAI-YT survey). These results showed that the eFlanker task effectively captures emotional biases in aging populations as well.

#### Online experiment

Participants in the online experiment performed the eFlanker and eGoNoGo tasks. Similar to the results from the young adults (in the offline experiment), we found a main effect of target emotion on RT in the eFlanker task (**Fig 3A**, RMANOVA, F(2,184) = 145.399, p<0.001), again, with a slower RT in the negative/positive conditions than the neutral condition (positive vs. neutral: t(92) = 12.821, p<0.001; negative vs. neutral: t(92) = 15.701, p<0.001, *Bonferroni corrected*). There was no effect of sex when it was added as a factor. Additionally, higher anxiety was generally associated with slower RT (Pearson r = 0.26, p = 0.012), and congruency effects in the higher anxiety group were marginally modulated by emotion (F(2, 88) = 2.978, p = 0.056).

Overall, the results from the generalizability tests indicated that an emotional bias in the eFlanker task was consistent across the lifespan, in depression patients, and across cultures and response modalities. Furthermore, negatively biased responses in the eFlanker and eGoNoGo tasks were associated with depression and anxiety.

### Extracting principal component “G-scores” from the emotional cognitive tasks

Because many behavioral indices from the three emotional cognitive tasks were highly correlated with one another, dimensionality reduction was conducted to convey precise and condensed information about individual behavioral patterns. Therefore, we calculated G-scores by grouping behavioral indices located closer in the low dimensional latent space. The behavioral data from the 264 participants, who performed all three cognitive tasks, including young adults and patients (but not online participants, children, and older adults, who only complete a subset of the tasks) were used for these analyses.

**Fig 4:**
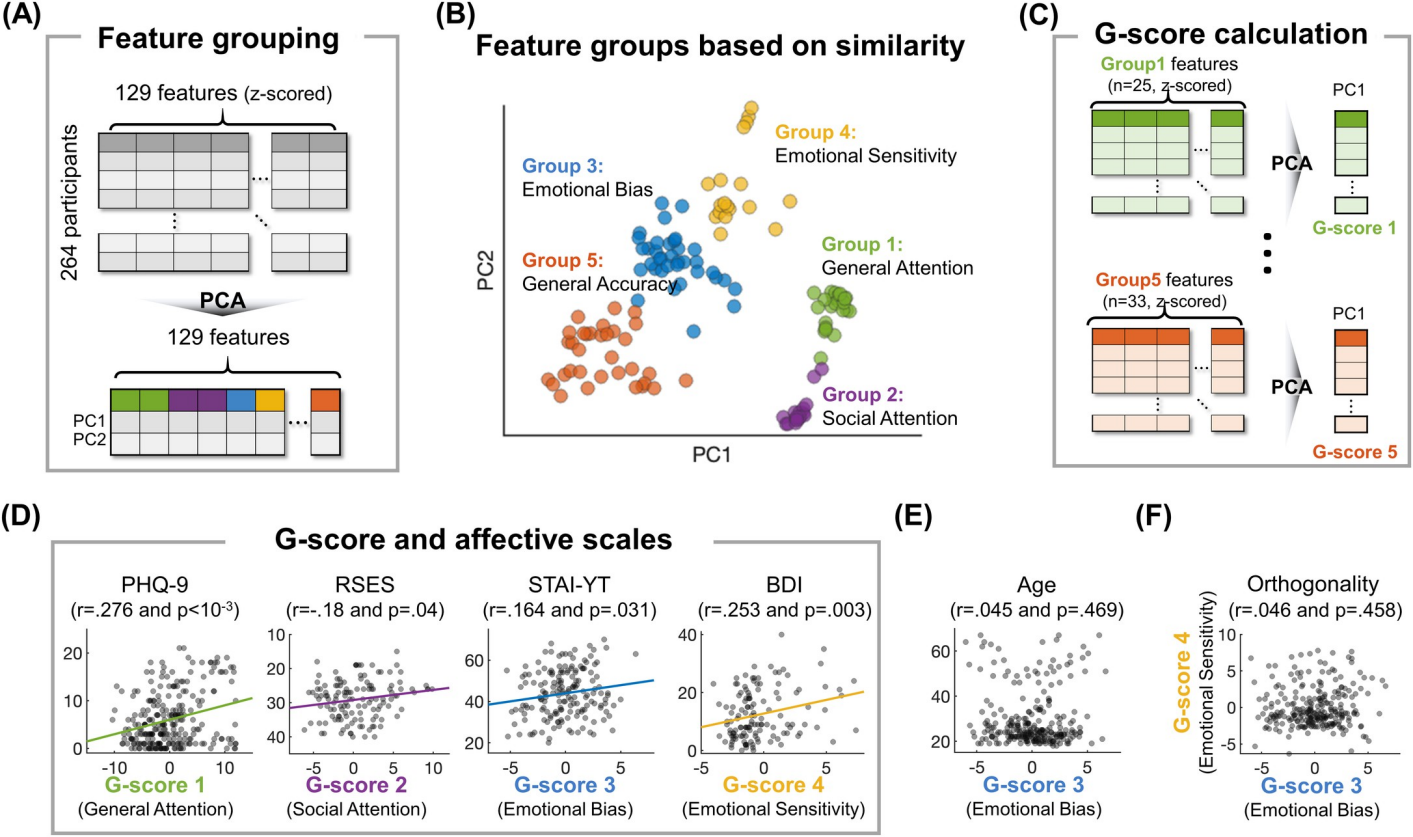
**(A)** Features were grouped based on their similarity across participants by PCA. **(B)** Feature grouping results visualized on a 2D plane for the first two principal components (PC) reveal five groups with different emotional cognitive characteristics. **(C)** G-scores were calculated by identifying the first PC from each feature group. **(D)** Correlations between each G-score and mental health scales. **(E)** G-score 3, which was indicative of a cognitive bias in processing emotional stimuli, was not correlated with age (See Table 3 for details). **(F)** While both G-scores 3 and 4 represented emotional effects, they were not correlated.

After applying the PCA, 129 behavioral features were divided into 5 groups based on the first two principal scores (**Fig 4A and 4B**). The first two feature groups contained RTs in various conditions of eFlanker & eGoNoGo and eSocial tasks, respectively, representing attention function. G-scores were calculated as the first PC from each feature group (**Fig 4C,** see Table 2 for the whole list of behavioral features and coefficients) and defined as the “General Attention” score (G-score 1) and the “Social Attention” score (G-score 2). Positive PC coefficients of RT features (Table 2) indicated that higher G-scores represented a reduction in attention-based performance.

**Table 2:** A list of behavioral indices from the emotional cognitive tasks and their grouping results along with coefficients for calculating the first principal component score.

NO.	Task	Feature	Group	Coeff.	NO.	Task	Feature	Group	Coeff.
1	eFlanker	RT overall	1	0.22	66	eSocial	RT push T: neg	2	0.24
2	eFlanker	RT T: pos / D: pos	1	0.21	67	eSocial	RT push T: nt	2	0.23
3	eFlanker	RT T: pos / D: neu	1	0.21	68	eSocial	RT push T: emo	2	0.25
4	eFlanker	RT T: pos / D: neg	1	0.2	69	eSocial	RT push T: all	2	0.25
5	eFlanker	RT T: neu / D: pos	1	0.19	70	eSocial	RT pull T: pos	2	0.24
6	eFlanker	RT T: neu / D: neu	1	0.2	71	eSocial	RT pull T: neu	2	0.24
7	eFlanker	RT T: neu / D: neg	1	0.19	72	eSocial	RT pull T: neg	2	0.24
8	eFlanker	RT T: neg / D: pos	1	0.2	73	eSocial	RT pull T: nt	1	0.15
9	eFlanker	RT T: neg / D: neu	1	0.2	74	eSocial	RT pull T: emo	2	0.25
10	eFlanker	RT T: neg / D: neg	1	0.2	75	eSocial	RT pull T: all	2	0.24
11	eFlanker	RT T: emo / D: cong	1	0.21	76	eSocial	RT push&pull T: pos	2	0.25
12	eFlanker	RT T: emo / D: incong	1	0.21	77	eSocial	RT push&pull T: neu	2	0.24
13	eFlanker	ΔRT (T:neg/D:neg) ‐ (T:pos/D:pos)	3	0.04	78	eSocial	RT push&pull T: neg	2	0.25
14	eFlanker	ΔRT (T:pos/D:pos) ‐ (T:neu/D:neu)	4	0.1	79	eSocial	RT push&pull T: nt	2	0.24
15	eFlanker	ΔRT (T:neg/D:neg) ‐ (T:neu/D:neu)	4	0.08	80	eSocial	RT push&pull T: emo	2	0.25
16	eFlanker	ΔRT (T:emo/D:cong) ‐ (T:neu/D:neu)	4	0.1	81	eSocial	ΔRT push (T:pos)-(T:neg)	3	0.07
17	eFlanker	RT T: pos / D: incong	1	0.2	82	eSocial	ΔRT push (T:pos)-(T:neu)	3	-0.19
18	eFlanker	RT T: neu / D: incong	1	0.19	83	eSocial	ΔRT push (T:neu)-(T:neg)	3	0.23
19	eFlanker	RT T: neg / D: incong	1	0.21	84	eSocial	ΔRT push (T:emo)-(T:neu)	3	-0.23
20	eFlanker	ΔRT (T:pos/D:incong)-(T:pos/D:cong)	3	0.03	85	eSocial	ΔRT push (T:pos)-(T:nt)	5	0.11
21	eFlanker	ΔRT (T:neu/D:incong)-(T:neu/D:cong)	3	-0.04	86	eSocial	ΔRT push (T:neu)-(T:nt)	5	0.07
22	eFlanker	ΔRT (T:neg/D:incong)-(T:neg/D:cong)	3	-0.03	87	eSocial	ΔRT push (T:neg)-(T:nt)	5	0.12
23	eFlanker	ΔRT (T:emo/D:incong)-(T:emo/D:cong)	3	0.01	88	eSocial	ΔRT push (T:emo)-(T:nt)	5	0.12
24	eFlanker	RT T: all / D: cong	1	0.22	89	eSocial	ΔRT pull (T:neg)-(T:pos)	3	0.05
25	eFlanker	RT T: all / D: incong	1	0.22	90	eSocial	ΔRT pull (T:pos)-(T:neu)	3	-0.14
26	eFlanker	ΔRT (T:all/D:incong)-(T:all/D:cong)	3	-0.03	91	eSocial	ΔRT pull (T:neg)-(T:neu)	3	-0.07
27	eFlanker	RT T: pos / D: all	1	0.21	92	eSocial	ΔRT pull (T:emo)-(T:neu)	3	-0.12
28	eFlanker	RT T: neu / D: all	1	0.19	93	eSocial	ΔRT pull (T:pos)-(T:nt)	5	0.15
29	eFlanker	RT T: neg / D: all	1	0.21	94	eSocial	ΔRT pull (T:neu)-(T:nt)	5	0.13
30	eFlanker	RT T: emo / D: all	1	0.21	95	eSocial	ΔRT pull (T:neg)-(T:nt)	5	0.13
31	eFlanker	ΔRT (T:pos/D:all)-(T:neu/D:all)	4	0.12	96	eSocial	ΔRT pull (T:emo)-(T:nt)	5	0.15
32	eFlanker	ΔRT (T:neg/D:all)-(T:neu/D:all)	4	0.1	97	eSocial	ΔRT push (T:all) ‐ pull (T:all)	3	0.01
33	eFlanker	ΔRT (T:neg/D:all)-(T:pos/D:all)	3	0.02	98	eSocial	ΔRT (T:neg)-(T:pos)	3	0.01
34	eFlanker	ΔRT (T:emo/D:all)-(T:neu/D:all)	4	0.12	99	eSocial	ΔRT (T:pos)-(T:neu)	3	-0.24
35	eFlanker	ACC overall	5	0.23	100	eSocial	ΔRT (T:neg)-(T:neu)	3	-0.23
36	eFlanker	ACC T: emo / D: cong	5	0.18	101	eSocial	ΔRT (T:emo)-(T:neu)	3	-0.26
37	eFlanker	ACC T: emo / D: incong	5	0.19	102	eSocial	ACC overall	5	0.25
38	eFlanker	ΔACC (T:neg/D:neg)-(T:pos/D:pos)	3	-0.01	103	eSocial	ACC push T:pos	5	0.18
39	eFlanker	ΔACC (T:emo/D:cong)-(T:neu/D:neu)	5	0.13	104	eSocial	ACC push T:neg	5	0.13
40	eFlanker	ACC T:pos / D: incong	5	0.2	105	eSocial	ACC push T:nt	5	0.17
41	eFlanker	ACC T:neg / D: incong	5	0.15	106	eSocial	ACC push T:emo	5	0.18
42	eFlanker	ΔACC (T:pos/D:incong)-(T:pos/D:cong)	3	-0.06	107	eSocial	ACC push T:all	5	0.22
43	eFlanker	ΔACC (T:neg/D:incong)-(T:neg/D:cong)	3	-0.02	108	eSocial	ACC pull T:all	5	0.22
44	eFlanker	ΔACC (T:emo/D:incong)-(T:emo/D:cong)	3	-0.03	109	eSocial	ACC T: pos	5	0.2
45	eFlanker	ACC T: all / D: cong	5	0.15	110	eSocial	ACC T: neg	5	0.19
46	eFlanker	ACC T: all / D: incong	5	0.21	111	eSocial	ACC T: nt	5	0.2
47	eFlanker	ΔACC (T:all/D:incong)-(T:all/D:cong)	3	-0.06	112	eSocial	ACC T: emo	5	0.22
48	eFlanker	ACC T: pos / D: target	5	0.19	113	eSocial	ΔACC push (T:pos)-(T:neg)	3	-0.05
49	eFlanker	ACC T: neg / D: target	5	0.16	114	eSocial	ΔACC push (T:pos)-(T:neu)	3	-0.35
50	eFlanker	ACC T: emo / D: target	5	0.23	115	eSocial	ΔACC push (T:neu)-(T:neg)	3	0.3
51	eFlanker	ΔACC (T:pos/D:all)-(T:neu/D:all)	5	0.16	116	eSocial	ΔACC push (T:emo)-(T:neu)	3	-0.34
52	eFlanker	ΔACC (T:neg/D:all)-(T:neu/D:all)	5	0.12	117	eSocial	ΔACC push (T:pos)-(T:nt)	4	0.25
53	eFlanker	ΔACC (T:neg/D:all)-(T:pos/D:all)	4	0.12	118	eSocial	ΔACC push (T:neu)-(T:nt)	4	0.24
54	eFlanker	ΔACC (T:emo/D:all)-(T:neu/D:all)	5	0.16	119	eSocial	ΔACC push (T:neg)-(T:nt)	4	0.27
55	eGoNoGo	RT overall	1	0.17	120	eSocial	ΔACC push (T:emo)-(T:nt)	4	0.27
56	eGoNoGo	RT T: pos	1	0.17	121	eSocial	ΔACC pull (T:pos)-(T:nt)	4	0.38
57	eGoNoGo	RT T: neg	1	0.16	122	eSocial	ΔACC pull (T:neu)-(T:nt)	4	0.37
58	eGoNoGo	ΔRT (T:neg)-(T:pos)	3	0.05	123	eSocial	ΔACC pull (T:neg)-(T:nt)	4	0.36
59	eGoNoGo	ERR overall	4	0.21	124	eSocial	ΔACC pull (T:emo)-(T:nt)	4	0.4
60	eGoNoGo	ERR T: pos	4	0.15	125	eSocial	ΔACC push (T:all) ‐ pull (T:all)	3	-0.01
61	eGoNoGo	ERR T: neg	4	0.15	126	eSocial	ΔACC push&pull (T:neg)-(T:pos)	3	0.02
62	eGoNoGo	ΔERR (T:neg)-(T:pos)	3	-0.02	127	eSocial	ΔACC push&pull (T:pos)-(T:neu)	3	-0.29
63	eSocial	RT overall	2	0.25	128	eSocial	ΔACC push&pull (T:neg)-(T:neu)	3	-0.33
64	eSocial	RT push T: pos	2	0.23	129	eSocial	ΔACC push&pull (T:emo)-(T:neu)	3	-0.31
65	eSocial	RT push T: neu	2	0.24					

**RT**: reaction time, **ACC**: accuracy, **ERR**: error rate

Δ: difference between two conditions

**T**: target, **D**: distractor

**pos**: positive, **neg**: negative, **neu**: neutral, **emo**: emotional (pos or neg), **nt**: non-target

**cong**: congruent (i.e. target = distractor), **incong**: incongruent (i.e. target ≠ distractor)

The third feature group represented complex interactions between emotion and attention. Coefficients of the first PC indicated that a higher G-score 3 was associated with a higher eFlanker congruency effect in the positive condition and a lower congruency effect in the negative/neutral conditions. Additionally, in the eSocial task, a higher G-score 3 indicated more negative/positive biases in the push/pull conditions, respectively (i.e. negative emotion induced slower RT in the pull condition but faster RT in the push condition). Furthermore, lower accuracy in the positive condition compared to the other conditions was associated with a higher G-score 3. Therefore, we defined G-score 3 as the “Emotional Bias” score.

The fourth group consisted mostly of RT differences between emotional and neutral conditions in the eFlanker RT or accuracy differences between emotional conditions and the non-target condition in the eSocial task. The first PC was defined as the “Emotional Sensitivity” score (G-score 4). Positive coefficients in calculating the score indicated that participants with a higher Emotional Sensitivity score took a longer time processing emotional stimuli but showed higher accuracy in the emotional conditions compared to the neutral or non-target conditions. Interestingly, the Emotion Bias and Emotion Sensitivity scores (G-score 3 and G-score 4) were not correlated (**Fig 4F**, Pearson r = 0.046 and p = 0.458). The fifth group was defined as the “General Accuracy” score (G-score 5) and was positively correlated with accuracy in the eFlanker and eSocial tasks.

We measured relationships between the G-scores and affective scales and found that each G-score was able to predict different individual affective states. (**Fig 4D**). General Attention was positively related to various mental health scales (e.g. STAI-YT, STAI-YS, BDI-II, PHQ-9, RSES, etc. See Table 3 for the whole list) which indicated that anxiety and depression were associated with declined attentional function. Social Attention was correlated only with self-esteem (RSES), which was consistent with the socially evaluative nature of the eSocial task. Although Emotional Bias and Emotional Sensitivity both represented emotion-attention interactions, the Emotional Bias score was associated with anxiety (STAI-YT), while Emotional Sensitivity score was associated with depression (BDI-II and PHQ-9) and self-esteem (RSES). General Accuracy score was not associated with any of the mental health scales. These results were significant after controlling for the effects of age (**Fig 4E** and Table 3).

Self-relevance/valence ratings from the spontaneous thought generation task were positively correlated with General Attention (S4 Fig, valence: r = 0.268, p = 0.001, self-relevance: r = 0.203, p = 0.014) but negatively correlated with General Accuracy (valence: r = -0.295, p<0.001, self-relevance: r = -0.16, p = 0.053). A weak association between behavioral indices related to emotion-attention interaction and the spontaneous thought generation task implied that the spontaneous thought generation task was a good indicator of mental health but largely independent of the cognitive functions extracted from our analysis.

**Table 3:** Partial correlations between G-scores and affective scales (controlling for age).

		PHQ-9	CES-D	BDI-II	RSES	STAI-YT	STAI-YS	Age
**G-score 1**	General Attention	r = .137* (p = .026)	r = .109 (p = .156)	r = .194* (p = .027)	r = -.213* (p = .015)	r = .232** (p = .002)	r = .239** (p = .002)	r = .646*** (p<10^−3^)
**G-score 2**	Social Attention	r = -.013 (p = .83)	r = .038 (p = .621)	r = .044 (p = .614)	r = -.172* (p = .05)	r = .076 (p = .32)	r = .025 (p = .744)	r = .498*** (p<10^−3^)
**G-score 3**	Emotional Bias	r = .015 (p = .80)	r = .124 (p = .105)	r = .126 (p = .153)	r = -.088 (p = .32)	r = .161* (p = .035)	r = .147 (p = .055)	r = .045 (p = .469)
**G-score 4**	Emotional Sensitivity	r = .149* (p = .016)	r = .064 (p = .406)	r = .239** (p = .006)	r = -.187* (p = .033)	r = .121 (p = .113)	r = .103 (p = .179)	r = .236*** (p<10^−3^)
**G-score 5**	General Accuracy	r = -.056 (p = .366)	r = .057 (p = .456)	r = -.065 (p = .461)	r = -.002 (p = .98)	r = -.027 (p = .723)	r = -.053 (p = .488)	r = -.367*** (p<10^−3^)

Overall, the G-scores concisely summarized a wide range of behavioral indices from the emotional cognitive tasks and were generally robust to covarying factors such as age, thereby providing a more precise measure of specific cognitive function and its interaction with emotion, for the purposes of evaluating and predicting individual affective scales.

### Training effects on task performance were associated with alleviated anxiety state

**Fig 5:**
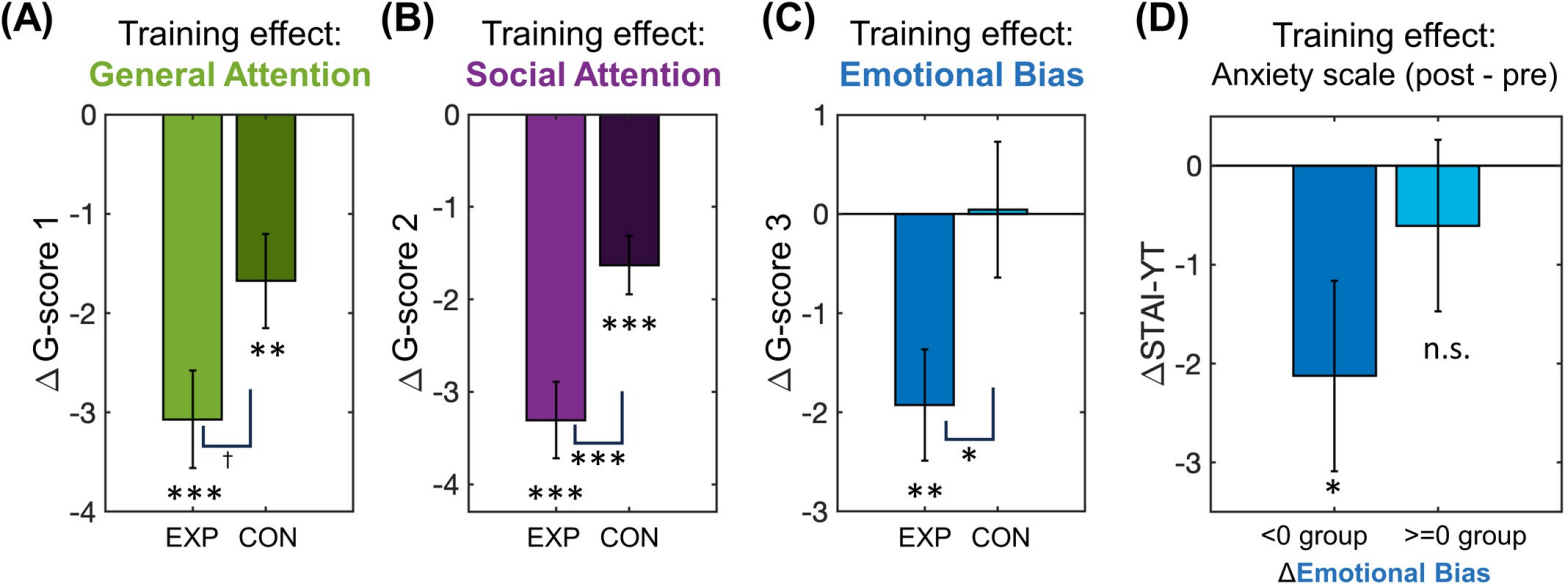
Training effects on G-scores. **(A-C)** A comparison of training effects between experimental (EXP) and control (CON) groups. Emotional Sensitivity and General Accuracy did not show training effects for both groups. **(D)** Only participants with decreased Emotional Bias after the training showed a significant decrease in anxiety scales (STAI-YT and STAI-YS (not shown)).

Healthy young adult participants (N = 71, N = 43 for the training group and N = 28 for the control group) underwent 2 weeks of training playing a gamified version of the emotional cognitive tasks (**Fig 1B** and S1 Video). To characterize the effects of the emotional training, we compared G-scores from pre- and post-training sessions (**Fig 5**). Significant differences between pre- and post-training were found in the General and Social Attention scores as well as the Emotional Bias score. First, we observed a decrease in the General and Social Attention scores, which represent faster RT for both control and experimental groups (**Fig 5A and 5B,** t-test between pre- and post-sessions for the experimental group: General Attention t(42) = -6.26, p<0.001, Social Attention t(42) = -8.03, p<0.001; control group: General Attention t(26) = -3.55, p = 0.002, Social Attention t(26) = -5.159, p<0.001). Nevertheless, the experimental group showed a larger decrease in both scores (t-test between groups: General Attention t(68) = -1.925, p = 0.058, Social Attention t(68) = -2.904, p = 0.005). While this indicated an improvement in performance in the experimental group, this was likely to be a result of the fact that the gamified training was based on the relevant cognitive tasks.

More importantly, the Emotional Bias score also changed only for the experimental group (**Fig 5C,** t-test between pre- and post-sessions: experimental group: t(42) = -3.43, p = 0.001; control group: t(26) = 0.062, p = 0.951; t-test between groups: t(68) = 2.205, p = 0.031). Given that the baseline Emotional Bias score was positively correlated with the anxiety scales (i.e. STAI-YT and STAI-YS), its decrease after the training may be indicative of a positive change in mental health. Consistent with this interpretation, the decrease in the Emotional Bias score was associated with a decrease in anxiety (i.e. STAI-YT and STAI-YS difference between the post- and pre-training sessions) (**Fig 5D,** STAI-YT: t(39) = -2.16, p = 0.037; STAI-YS: t(39) = -2.784, p = 0.008). Emotional Sensitivity and General Accuracy scores did not show any training effects.

A difference in training effects between the 4-session and 6-session training groups was only observed for Social Attention, with the 6-session group showing a greater improvement in RT (RMANOVA interaction between pre-post change and training: F(1,67) = 4.939, p = 0.03).

### Resting-state EEG as a neural marker of emotional attention bias and its enhancement over gamified training

It is well known that neural activity in the prefrontal cortex plays an important role in cognition and emotional processing [70–72] and may be a meaningful biomarker underlying the individual differences in mental health [7,73,74]. Therefore, in order to verify whether our findings can be traced back to specific neural activity in the prefrontal cortex, we investigated the association between resting-state prefrontal scalp EEG markers with both mental health scales and behavioral performance on the emotional cognitive tasks.

**Fig 6:**
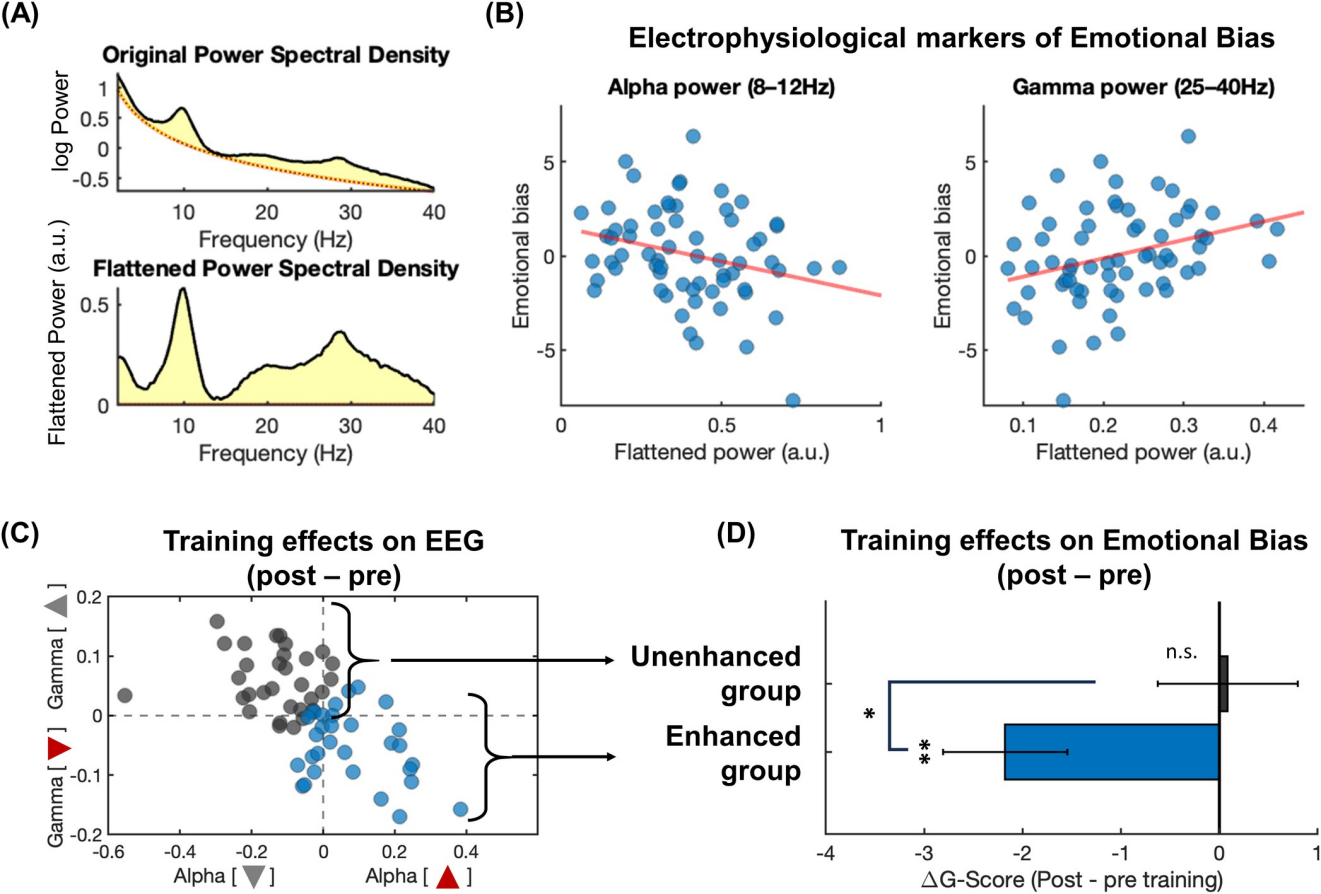
EEG markers of behavioral patterns in the emotional cognitive tasks. **(A)** An example of the original and flattened power spectral density. The flattened power spectrum was calculated with the FOOOF algorithm (see Methods) and used for further analyses. **(B)** Significant correlations between Emotional Bias and the alpha power (left, negative correlation) and gamma power (right, positive correlation) were observed. **(C)** A scatter plot of training effects (i.e. post-pre) on z-scored alpha and gamma power. Enhanced (blue) and Unenhanced (black) groups were defined based on the median split of the summed absolute magnitude of alpha power increase and gamma power decrease. A change in EEG in the direction of alpha decrease or gamma increase was correlated with an improvement in Emotional Bias. **(D)** Significant training effects on the Emotional Bias (G-score 3) were observed in the EEG enhanced group.

First, we dissociated neural oscillations from the aperiodic exponent (i.e. spectral slope) by applying the FOOOF algorithm [66] (**Fig 6A**). Aperiodic exponent was associated with depression (S5 Fig, CES-D, r = -0.297, p = 0.016); therefore, it was important to dissociate it from oscillatory power. After correcting for the aperiodic component, we found that alpha (8–12 Hz) and gamma (25–40 Hz) power were associated with the Emotional Bias score (**Fig 6B**): First, participants with lower alpha power showed higher Emotional Bias (r = -0.27, p = 0.029), suggesting that a less inhibited prefrontal cortex (as indicated by lower alpha power [75,76]) represents a more anxious, emotionally deregulated state. On the contrary, gamma (25–40 Hz) power was positively correlated with Emotional Bias (r = 0.313, p = 0.011), which is consistent with the above result in the alpha band, considering that gamma oscillations are widely thought to represent an excitatory neural state [77]. Notably, the alpha/gamma marker was specific to the interaction between cognition and emotion in that other oscillatory frequency bands and aperiodic exponent did not correlate with the Emotional Bias score (S6 Fig).

If resting-state alpha and gamma power are truly neural markers of Emotional Bias, then a training-related change in resting-state alpha/gamma should correspond to a change in the Emotional Bias score as well. We tested this possibility by defining the EEG training effect as the sum of the magnitude of the alpha increase and gamma decrease after z-score normalization of oscillatory power for each subject (**Fig 6C and 6D**). Participants were divided into two groups based on this EEG training effect (**Fig 6C**): the Enhanced group consisted of individuals who showed both alpha increase and gamma decrease (alpha: t(30) = 3.659, p = 0.001; gamma: t(30) = -4.831, p<0.001), while the Unenhanced group did not. Crucially, the change in neural activity from pre- to post-training was associated with the degree of improvement in the Emotional Bias score (**Fig 6D**). The Enhanced group with increased alpha and decreased gamma power (**Fig 6C**) showed a significantly greater decrease in their Emotional Bias (t-test between pre- and post-sessions: t(30) = -3.435, p = 0.002), and this was significantly different from the Unenhanced group (t(60) = -2.372, p = 0.021), whose Emotional Bias did not change (t(30) = 0.118, p = 0.97) (**Fig 6D**). These results provided a neuroscientifically rooted basis for explaining anxiety-related individual differences in emotional cognitive bias, as well as its potential as a marker for brain-based digital therapeutics for improving mental health and its effects on cognitive performance.

## Discussion

In this study, we developed a set of digital tasks using emotional graphics and animation and tested its validity, generalizability, and trainability of cognition and emotion processing. Behavioral indices of Emotional Bias not only served as indicators of higher levels of anxiety but also showed improvement as a result of our gamified digital training program. We were also able to identify resting-state prefrontal alpha and gamma power as neural correlates of Emotional Bias.

### Validity of the emotional cognitive tasks

The eFlanker, eGoNoGo, and eSocial tasks were designed to test how cognitive functions (i.e. attention, working memory, and social cognition) interacted with emotional stimuli and how this interaction was associated with individual affective mental health measures such as anxiety, depression, and self-esteem. These emotional cognitive tasks were validated by testing a wide range of participants including patients, children, and older adults. Generalized results were observed across different groups, especially with respect to the emotional bias observed in the participants with higher anxiety and depression. These tasks not only offer an effective monitoring of depression and anxiety that can benefit individuals across a wide age range, the consistent result from the online experiment show additional promise in their implementation as an at-home digital therapy.

We introduced a feature reduction technique in calculating G-scores to maximize our ability to find meaningful information from the various behavioral indices provided by the tasks, while minimizing issues of noisy data and collinearity across features that may obscure the interpretation of relationships between the features and dependent variables. Each G-score was able to predict different aspects of the mental state of participants (i.e., General Attention: anxiety and depression; Social Attention: self-esteem; Emotional Bias: anxiety; Emotional Sensitivity: depression and self-esteem). The fact that among the 5 G-scores, only the Emotional Bias score was associated with anxiety, improved through training, and associated with a specific neural marker speaks further to the specificity and orthogonality of our G-scores. From the perspective of creating digital therapeutic applications, these G-scores provided a summary of an individual’s performance in the form of a simple 5-factor cognitive-affective profile for providing users with feedback following a session of the task.

### Frontal EEG correlates of emotional bias

Using a simple, 2-channel wearable EEG headset that can be easily incorporated into a brain-based cognitive therapy program, we found that frontal alpha and gamma power stood out as useful markers of the interaction between emotion and attention. Emotional Bias was associated with a lower resting-state alpha and was accompanied by high gamma power. These EEG markers were also correlated with individual levels of anxiety.

Although these neural markers were measured during a period of inactivity before the execution of the cognitive tasks, resting-state alpha power has been reported to be correlated with activities in the frontal default mode network [78,79] and may represent internally oriented attentional control that can influence readiness for perception and subsequent actions [80]. High baseline levels of anxiety can disrupt this regulatory process which is required for optimal performance of goal-directed actions. On the other hand, gamma oscillations are generally thought to represent a neural excitatory state of the prefrontal cortex [77] and, in the context of emotional information processing, may be correlated with synchronization between the medial prefrontal cortex and basolateral amygdala [81,82] that may be crucial for emotional regulation[83]. Against the backdrop of such numerous findings on frontal alpha/gamma oscillations, their correlation with the Emotional Bias score in our study may be interpreted in terms of fronto-limbic attentional control and emotional processing mechanisms recruited in our digital cognitive affective tasks. The decrease in the Emotional Bias score in the training experiment further showed its potential as a target behavioral marker for the development of digital therapeutics for improving cognitive and affective health.

### Potential and limitations for cognitive training

Our training program using a gamified version of the cognitive tasks was effective in specifically reducing Emotional Bias and, at the same time, in alleviating anxiety. It is also important to note that the control group did not show such effects. However, to improve the effectiveness of the gamified training task, it is important to find the optimal duration and intensity of the training and to examine the potential for personalized interventions based on individual cognitive-affective profiles. For instance, two weeks of training may not be sufficient to determine the long-term effectiveness of the training tasks; assessing the durability of the observed improvements in Emotional Bias and anxiety over an extended period will be an important step towards the development of a cognitive-affective digital therapy application. At the same time, any adverse effect induced by long-term usage of gamified emotional training must also be tested, although the frequency of the training used in this study is not expected to induce any adverse effects in even children and older adults, based on previous studies [84].

The effectiveness of gamified emotional training can be improved in several ways based on detailed investigations. Given the design of this training study, the control group was instructed to perform a coloring, picture comparison, and visual search task. We chose these tasks in order to prevent unexpected training effects induced by a cognitively demanding control task; future studies may further address the domain specificity of our findings using other cognitive training tasks. For example, a control condition that closely matches the training condition but lacks the emotional component could help identify whether the observed effects could be attributed to attentional training, emotional training, or both.

### Implications for future research

In combination with the spontaneous thought generation task, which can be utilized as a first-order approximation of various mental health scales, such a gamified approach to cognitive affective digital therapy holds great promise for the future, particularly for children and older adults who may have difficulty with traditional cognitive behavioral therapy. In addition, we believe that the analysis techniques using dimensionality reduction and the neural measurement with wearable EEG can provide a starting point for scientifically validated brain-based digital therapeutic tools.

Because all of the participants in our training experiment were healthy individuals who reported to have never been diagnosed with any psychiatric disorders, future studies may benefit from more accurate self-report measures of mental health that can be verified through clinical assessment. Furthermore, the effects of training in healthy individuals should be compared and further validated with clinically diagnosed patient populations.

Going forward, the inclusion of a broader range of factors and individual data, including socioeconomic, educational, physical health, and environmental influences could provide a more comprehensive understanding of mental health and the potential benefits of digital gamified interventions. To that end, collaborative efforts with government agencies to collect large public data using such application-based tasks may be extremely effective in the advancement of digital therapeutics and healthcare in general.

## Supporting information

S1 Video Recorded video of playing the emotional digital game-based training program. https://youtu.be/-kDOWhXxi48
(DOCX)

S1 Table The number of trials for each condition and the practice session across participants. (PDF)

S1 Fig A diagram of the spontaneous thought generation task. (PDF)

S2 Fig Behavioral results from young adults in the eFlanker task (which are not shown in Fig 2). (A) *Left*: A comparison of RT across three emotional conditions (Pos: positive, Neu: neutral, and Neg: negative). *Right*: A comparison of RT between congruent and incongruent conditions in higher and lower depression groups. (B) Congruency effects on RT across three emotional conditions in higher and lower depression groups. (C) Congruency effects on RT across three emotional conditions in higher and lower anxiety groups.
(PDF)

S3 Fig Scatter plots for indicating the relationship between mental health (depression, anxiety, and self-esteem) and two behavioral measures from the spontaneous thought generation task (i.e. self-relevance and valence). (A) Pearson correlation coefficients r = 0.218, p = 0.007. (B) r = -0.051, p = 0.611. (C) r = -0.415, p<10^−3^. (D) r = -0.44, p<10^−3^. (E) r = 0.341, p<10^−3^.
(PDF)

S4 Fig Scatter plots for indicating a relationship between G-scores and two behavioral measures from the spontaneous thought generation task (i.e. self-relevance and valence). (A) Pearson correlation coefficient r = 0.203, p = 0.014 (B) r = 0.108, p = 0.193 (C) r = -0.153, p = 0.065 (D) r = 0.070, p = 0.399 (E) r = -0.160, p = 0.053 (F) r = 0.268, p = 0.001 (G) r = 0.222, p = 0.007 (H) r = 0.061, p = 0.465 (I) r = 0.040, p = 0.632 (J) r = -0.295, p<10^−3^.
(PDF)

S5 Fig A Pearson correlation coefficient between the averaged aperiodic exponent from two forehead EEG channels and the depression scale (CES-D) was significant (r = -0.297, p = 0.016). (PDF)

S6 Fig Unlike alpha and gamma power (Fig 6B), correlations between Emotional Bias and the aperiodic exponent (left), flattened theta power (middle), and flattened beta power (right) were not significant (theta: r = 0.066, p = 0.6; beta: r = -0.209, p = 0.095). (PDF)
