# Supplementary material for: Digital assessment of cognitive-affective biases related to mental health
Source: PLOS Digit Health. 2024 Aug 29;3(8):e0000595. doi: 10.1371/journal.pdig.0000595 (PMC11361731; doi:10.1371/journal.pdig.0000595)
Supplement: S1 Video — https://youtu.be/-kDOWhXxi48 (DOCX) [file pdig.0000595.s001.docx]

**S1 Video.** Recorded video of playing the emotional digital game-based training program. <https://youtu.be/-kDOWhXxi48>
