# Supplementary material for: Digital assessment of cognitive-affective biases related to mental health
Source: PLOS Digit Health. 2024 Aug 29;3(8):e0000595. doi: 10.1371/journal.pdig.0000595 (PMC11361731; doi:10.1371/journal.pdig.0000595)
Supplement: S1 Table — (PDF) [file pdig.0000595.s002.pdf]

Supplementary Table 1.

Tasks	Participants	Practice	Target	Non-target
eFlanker	Online & offline young adults, older adults	36	270 (30 trials per condition[a])	Not applicable
	Patients	36	144 (16 trials per condition[a])	Not applicable
	Children	18	180 (20 trials per condition[a])	Not applicable
eGoNoGo	Online & offline young adults	40	96 (48 Pos and 48 Neg)	144 (72 Pos and 72 Neg)
	Patients	40	56 (28 Pos and 28 Neg)	84 (42 Pos and 42 Neg)
	Children	20	48 (24 Pos and 24 Neg)	72 (36 Pos and 36 Neg)
eSocial	Offline young adults	4	150 (60 Pos, 30 Neu, and 60 Neg)	54
	Patients	4	90 (36 Pos, 18 Neu, and 36 Neg)	24

[a]: Combinations across positive (Pos), negative (Neg), and neutral (Neu).
