# Supplementary figures and images for: Digital assessment of cognitive-affective biases related to mental health
Source: PLOS Digit Health. 2024 Aug 29;3(8):e0000595. doi: 10.1371/journal.pdig.0000595 (PMC11361731; doi:10.1371/journal.pdig.0000595)

# Spontaneous Thought Generation Task

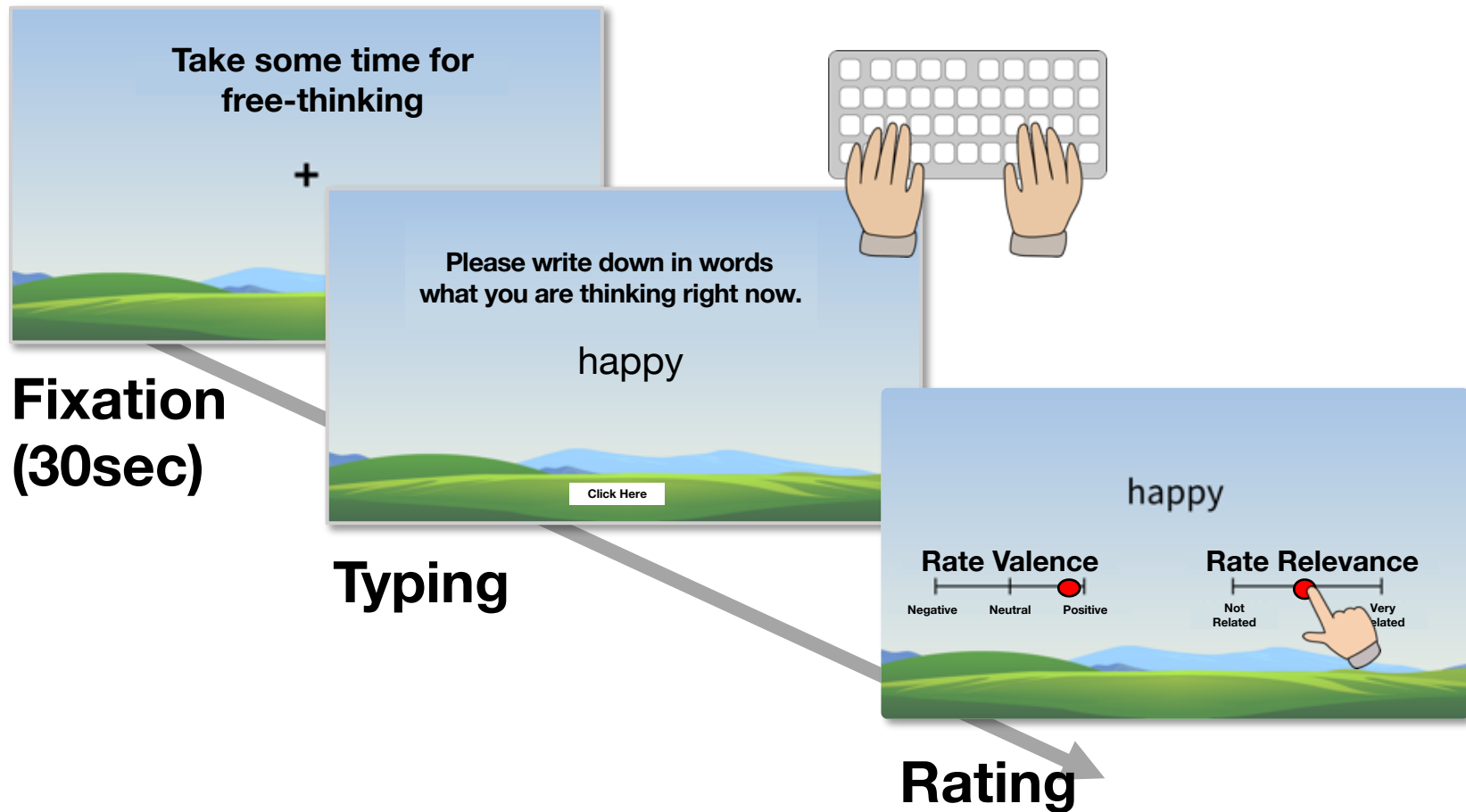

Supplement: S1 Fig — (PDF) [file pdig.0000595.s003.pdf]

**(A)**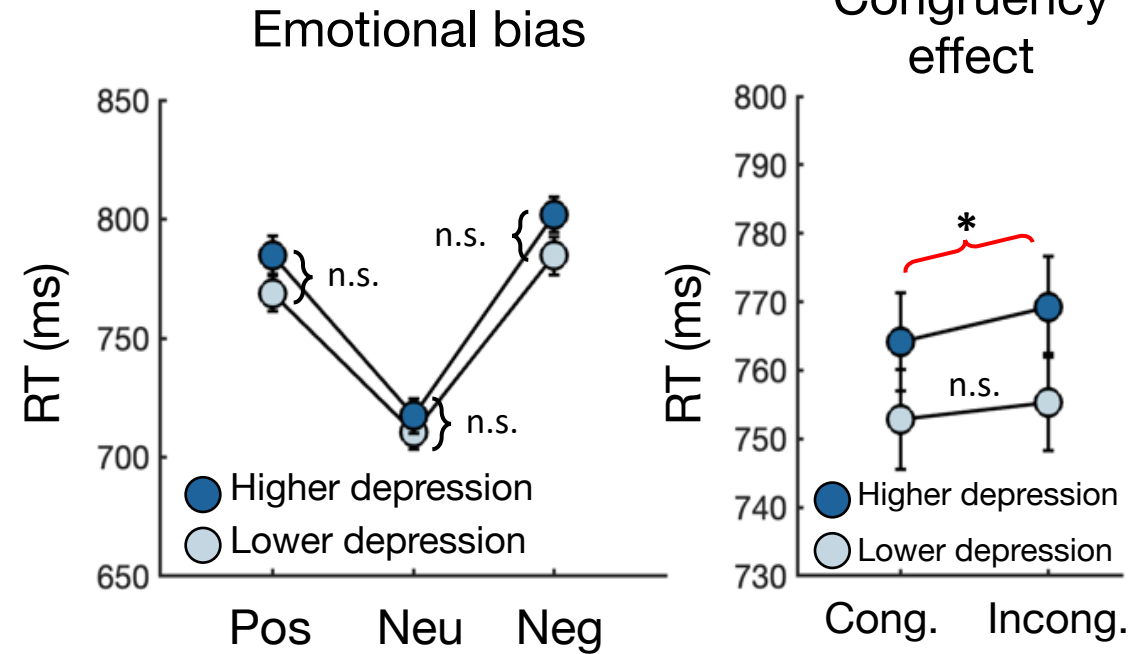**(B)**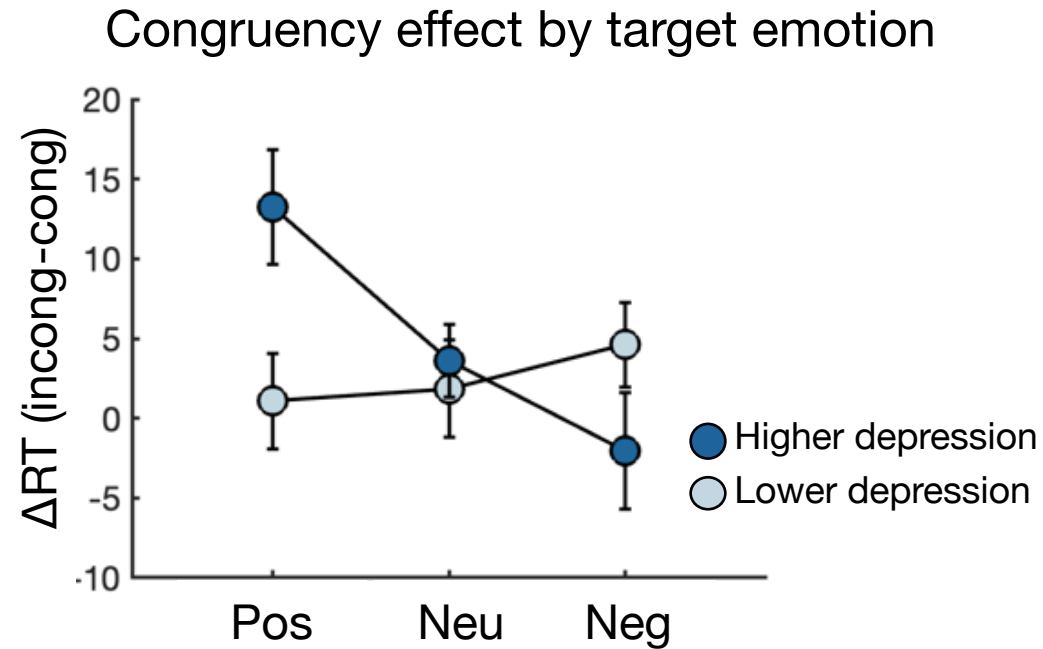**(C)**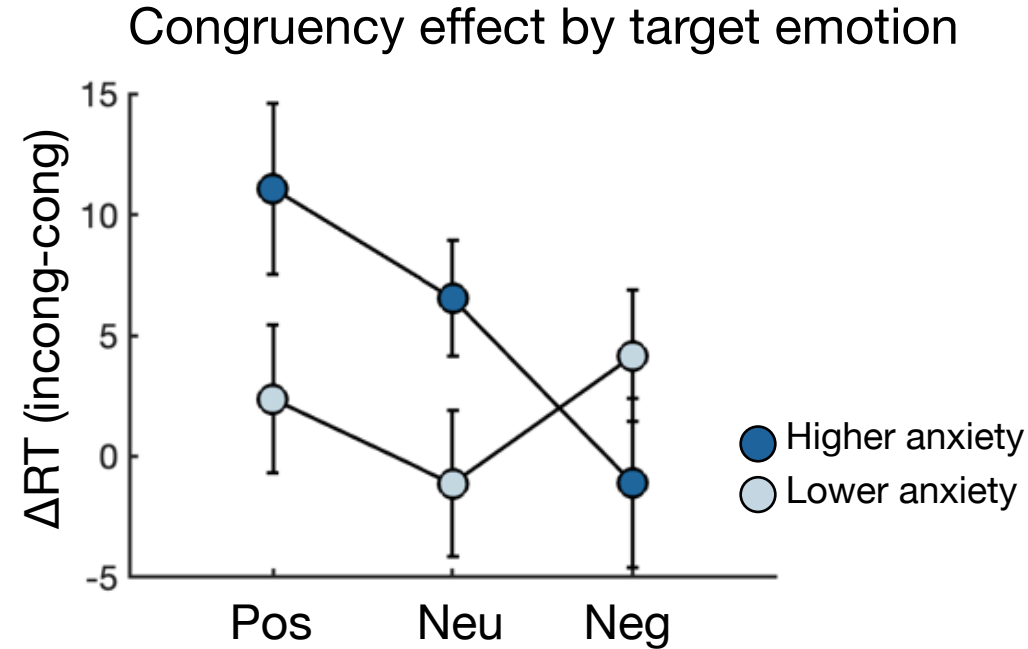

Supplement: S2 Fig — (A) Left: A comparison of RT across three emotional conditions (Pos: positive, Neu: neutral, and Neg: negative). Right: A comparison of RT between congruent and incongruent conditions in higher and lower depression groups. (B) Congruency effects on RT across three emotional conditions in higher and lower depression groups. (C) Congruency effects on RT across three emotional conditions in higher and lower anxiety groups. (PDF) [file pdig.0000595.s004.pdf]

**(A)**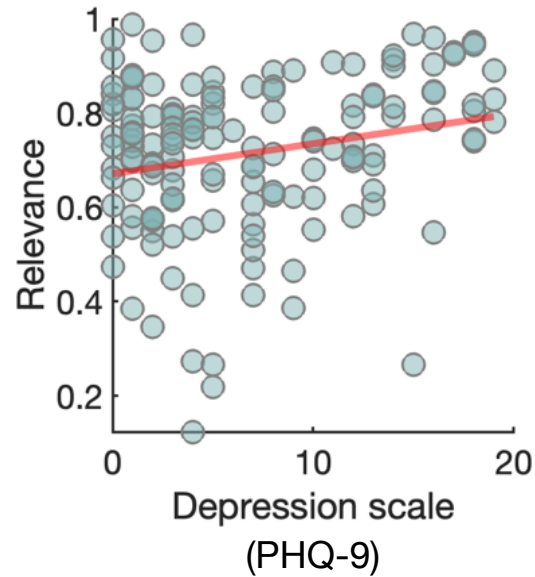**(B)**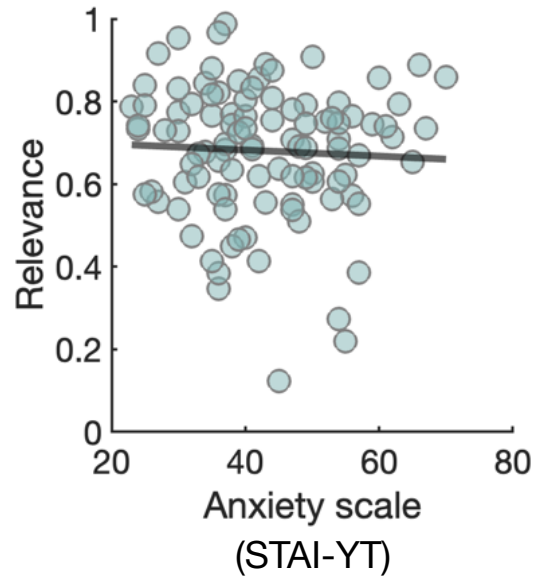**(C)**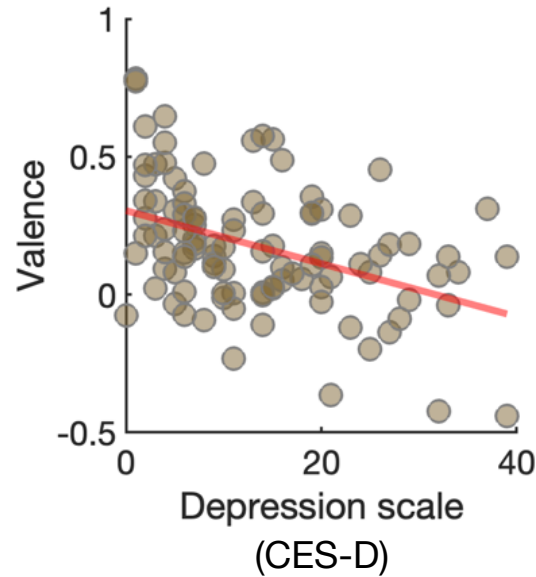**(D)**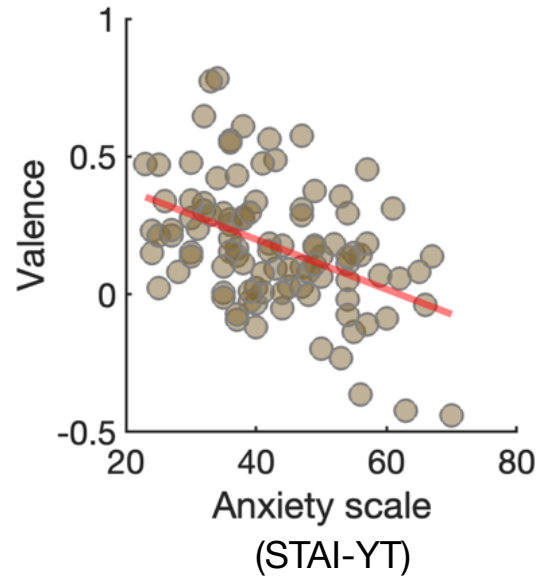**(E)**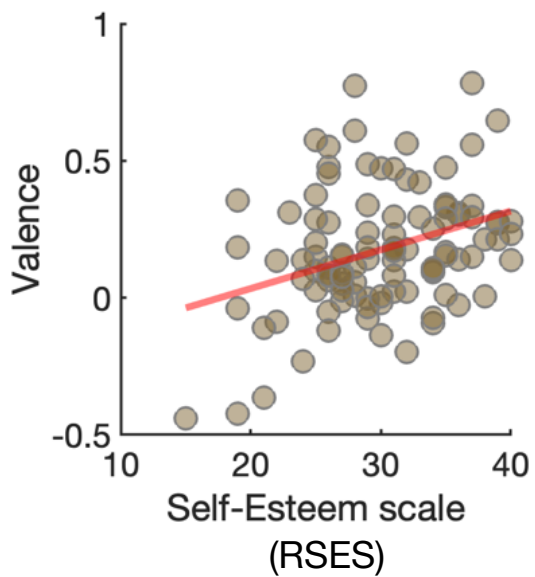

Supplement: S3 Fig — (A) Pearson correlation coefficients r = 0.218, p = 0.007. (B) r = -0.051, p = 0.611. (C) r = -0.415, p<10−3. (D) r = -0.44, p<10−3. (E) r = 0.341, p<10−3. (PDF) [file pdig.0000595.s005.pdf]

**(A)**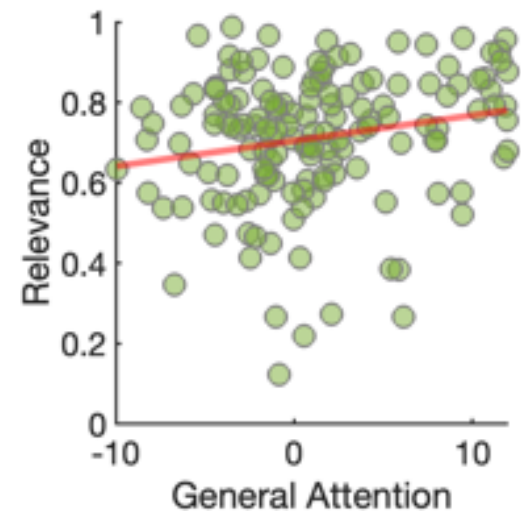**(B)**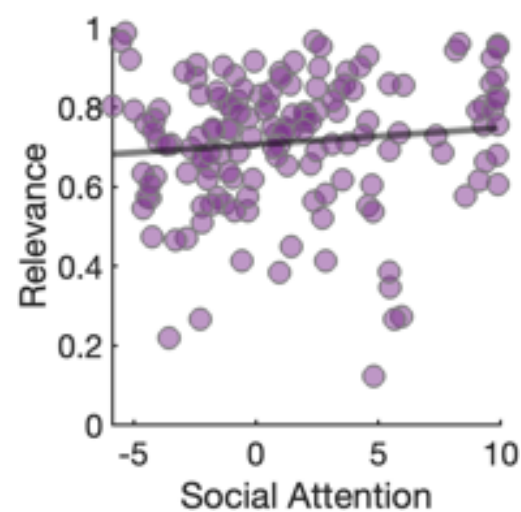**(C)**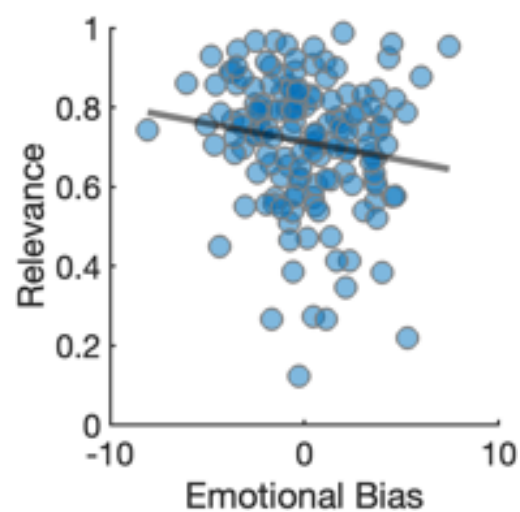**(D)**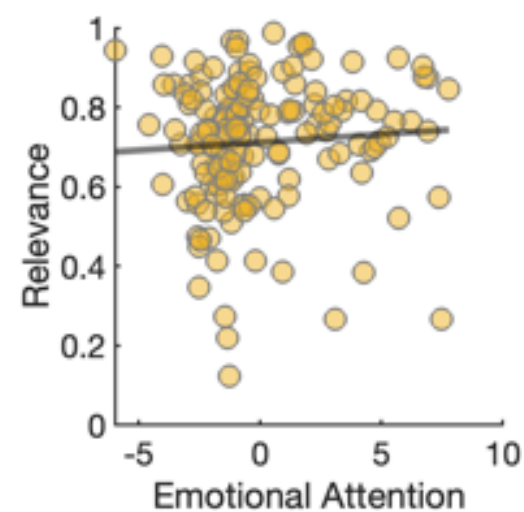**(E)**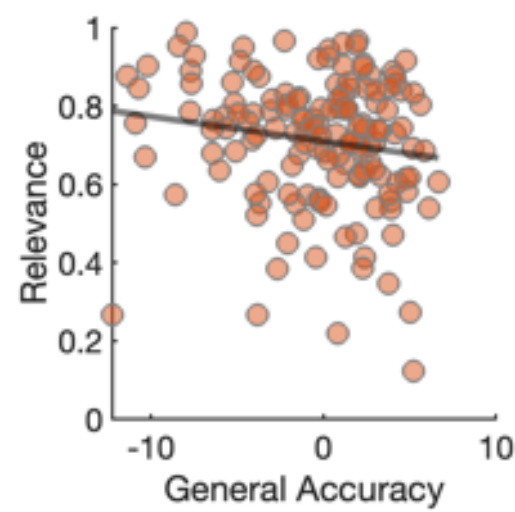**(F)**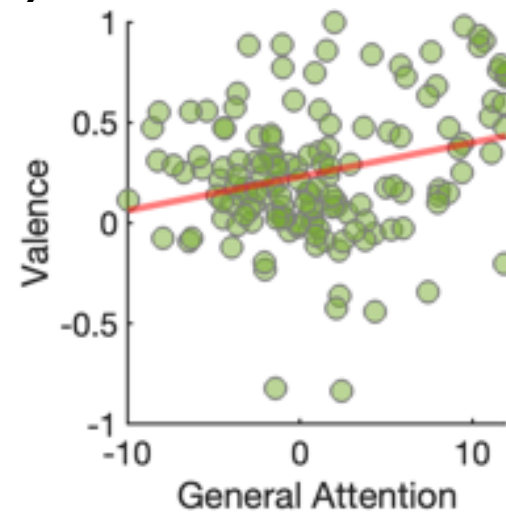**(G)**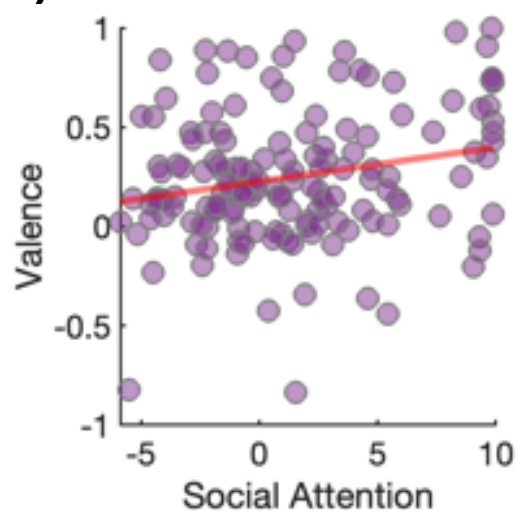**(H)**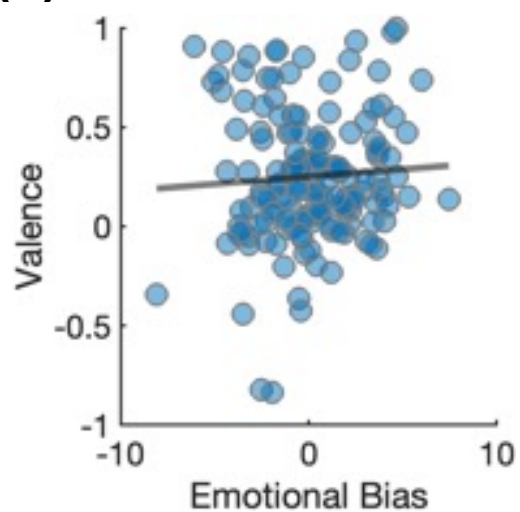**(I)**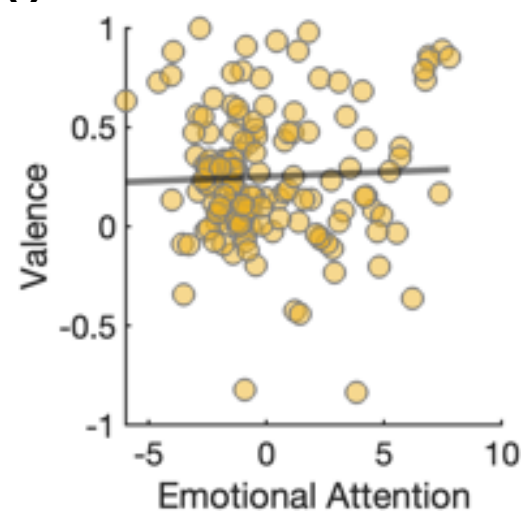**(J)**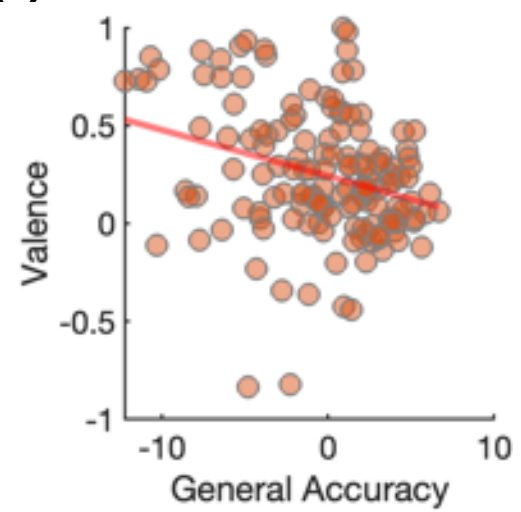

Supplement: S4 Fig — (A) Pearson correlation coefficient r = 0.203, p = 0.014 (B) r = 0.108, p = 0.193 (C) r = -0.153, p = 0.065 (D) r = 0.070, p = 0.399 (E) r = -0.160, p = 0.053 (F) r = 0.268, p = 0.001 (G) r = 0.222, p = 0.007 (H) r = 0.061, p = 0.465 (I) r = 0.040, p = 0.632 (J) r = -0.295, p<10−3. (PDF) [file pdig.0000595.s006.pdf]

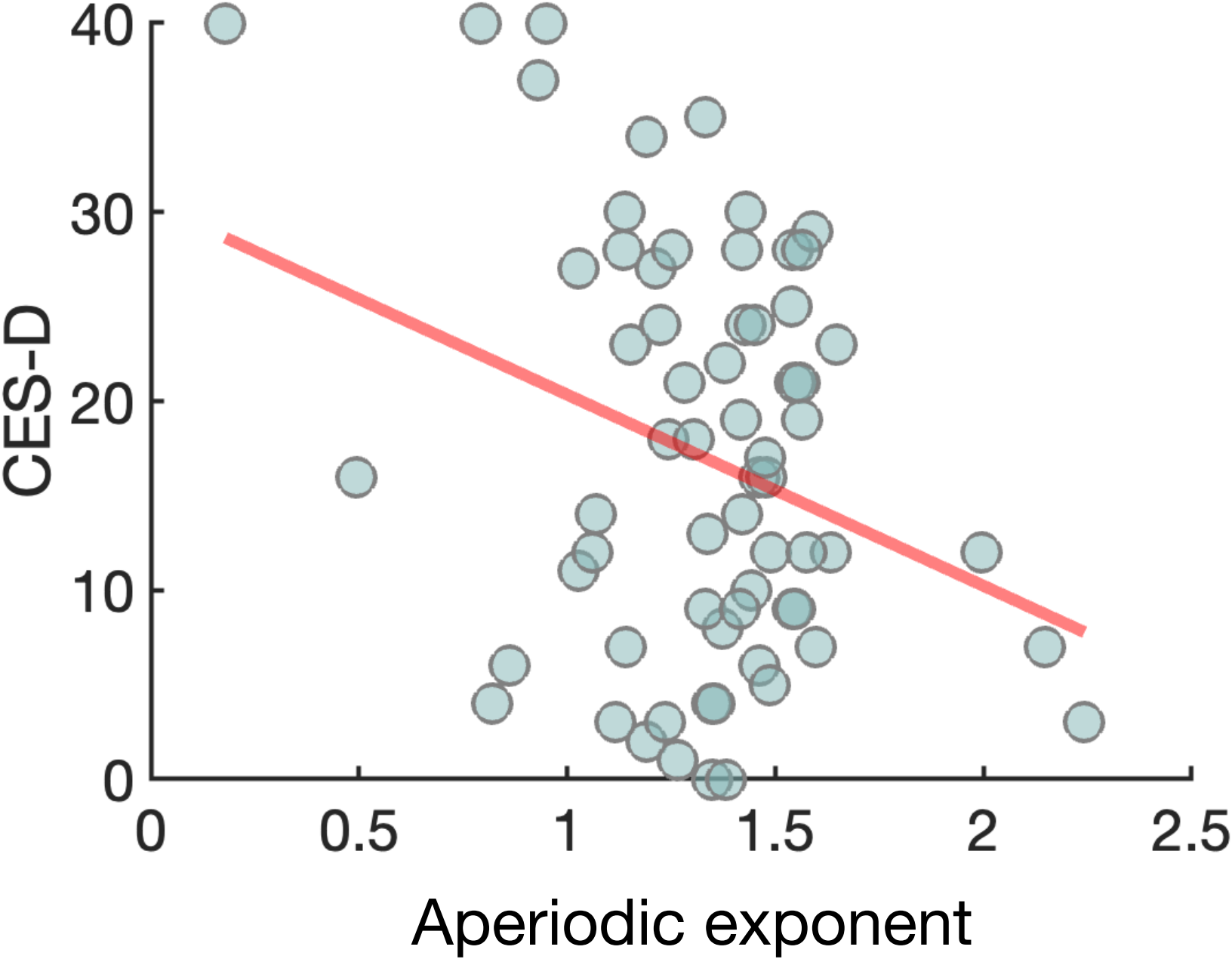

Supplement: S5 Fig — (PDF) [file pdig.0000595.s007.pdf]

**Aperiodic exponent**

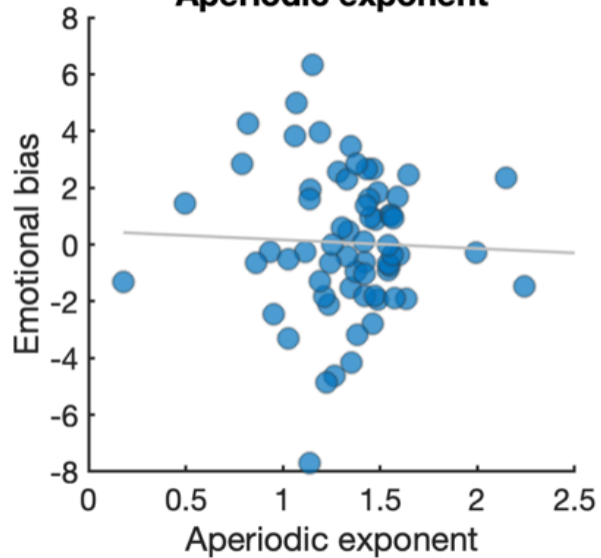

**Theta power (4–8Hz)**

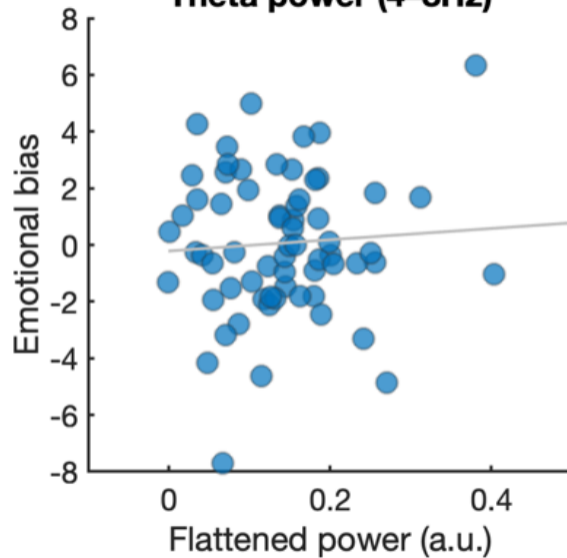

**Beta power (12–25Hz)**

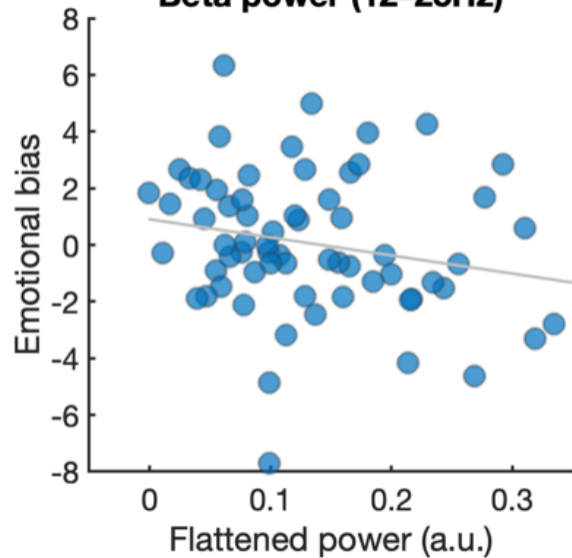

Supplement: S6 Fig — (PDF) [file pdig.0000595.s008.pdf]
